# Confidence, prediction, and tolerance in linear mixed models

**DOI:** 10.1002/sim.8386

**Published:** 2019-10-28

**Authors:** Bernard G. Francq, Dan Lin, Walter Hoyer

**Affiliations:** ^1^ TRD ‐ CMC Statistical Sciences GSK Rixensart Belgium; ^2^ Pre‐Clinical & Research ‐ Biostatistics and Statistical Programming GSK Rixensart Belgium; ^3^ TRD ‐ CMC Statistical Sciences GSK Marburg Germany

**Keywords:** assay validation and qualification, generalized Satterthwaite, Hessian and Fisher information matrix, intralesional resection, mixed model, prediction interval, tolerance interval

## Abstract

The literature about Prediction Interval (PI) and Tolerance Interval (TI) in linear mixed models is usually developed for specific designs, which is a main limitation to their use. This paper proposes to reformulate the two‐sided PI to be generalizable under a wide variety of designs (one random factor, nested and crossed designs for multiple random factors, and balanced or unbalanced designs). This new methodology is based on the Hessian matrix, namely, the inverse of (observed) Fisher Information matrix, and is built with a cell mean model. The degrees of freedom for the total variance are calculated with the generalized Satterthwaite method and compared to the Kenward‐Roger's degrees of freedom for fixed effects. Construction of two‐sided TIs are also detailed with one random factor, and two nested and two crossed random variables. An extensive simulation study is carried out to compare the widths and coverage probabilities of Confidence Intervals (CI), PIs, and TIs to their nominal levels. It shows excellent coverage whatever the design and the sample size are. Finally, these CIs, PIs, and TIs are applied to two real data sets: one from orthopedic surgery study (intralesional resection risk) and the other from assay validation study during vaccine development.

## 1 INTRODUCTION

Throughout medical and pharmaceutical research, linear mixed models are frequently used. When investigating the effect of new drugs, statistical reports and medical papers usually report the estimated treatment effects with their corresponding Confidence Intervals (CIs). CIs are typically used to assess the uncertainty on an unknown population parameter, eg, the estimated average response for a given population. During clinical studies, the average difference between population means in different study arms is of key interest, while random factors may need to be accounted for when observations are not independent. For release of a pharmaceutical product, on the other hand, suitable analytical release methods for impurities and the active ingredient need to be developed and qualified. The manufacturer needs to be confident that the measured release value is within a given percentage of the true (antigen or impurity) concentration. During an analytical method qualification, the bias (the systematic error, related to the trueness) and the random variability (related to the precision) are assessed. The accuracy of an analytical method describes the combined effects of trueness and precision and contains the effect of bias and variability on individual release values. During analytical method qualification, it is not only important to investigate the systematic bias of the analytical method but also the ranges within which individual release values will deviate from their true amount. In an assay qualification study during vaccine development, the trueness is evaluated using the CIs for the mean method bias at each concentration of the sample. On the other hand, Prediction Intervals (PIs) are very useful to assess the range of values within which a future observation may lie, eg, where a measurement of concentration is expected to lie in a new batch. Note that a PI can also be named as Tolerance Interval (TI) type I or *β*‐expectation TI. Furthermore, a confidence level can be added to a PI, to obtain a TI type II (or beta‐gamma content). TIs are therefore a good extension to PIs especially for small sample sizes, as the uncertainty of estimating the quantiles with a limited number of observations is taken into account. These intervals are applied in many contexts, especially in pharmaceutical industries,^1^, ^2^ and more generally in industrial statistics (see the book from Kenett et al^1^). TIs can also be used for process control in both the univariate and multivariate cases, as already proposed by Fuchs and Kenett^3^ in the eighties. In method comparison studies (also called bridging, eg, for analytical method transfer between laboratory or reagent bridging of critical reagents), the prediction (or tolerance) intervals are used to assess the range of the differences between two clinical or analytical measurement methods.^4^, ^5^ It has been recently shown that PIs and TIs are better than agreement intervals to compare two clinical measurement methods.^4^ In transfer of an analytical procedure, individual future values in a receiving laboratory should be “similar” to those of the reference laboratory. In assay or analytical method qualification, the accuracy is related to the PI when observed concentrations are compared to their theoretical known values. The PI or TI for future measures should, ideally, lie completely inside an equivalence margin, eg, the future measurements will not differ more than 20% from their nominal level.

The concepts of CI, PI, and TI will be here revisited under the framework of linear mixed models, including fixed effects of any type. The advantages, the coverage probabilities, and convergence of these intervals will be discussed. The literature usually focuses on one‐way random models (see overview by Krishnamoorthy and Mathew^6^), random effects models,^6^, ^7^, ^8^ or Monte Carlo simulation^9^ to calculate the TIs. These formulas are no longer appropriate in case of unbalanced or more complex designs, and this is a main limitation to their use. Furthermore, most of the papers deal with one‐sided intervals (see the introductory overview by Sharma and Mathew^10^), while two‐sided intervals will be the main focus of this paper. On the other hand, Sharma and Mathew^10^ proposed a mathematical approach based on small‐sample asymptotic properties to calculate one‐sided or two‐sided TIs for balanced or unbalanced designs. This methodology will be here compared to the modified large sample (MLS) approach applied on the expected mean squares (EMS) for balanced or unbalanced designs.

We propose a novel analytical two‐sided PI formula that is generalizable under a wide variety of designs (one random factor, multiple random factors with nested or crossed structure, and balanced or unbalanced designs). The generalized Satterthwaite's method, based on the Hessian matrix, calculates the degrees of freedom for the variance of the prediction. The sum of the variances for fixed effects and variance components is directly used as the variance for the prediction. This approach is compared to the classical Satterthwaite approximation on the mean squares for the degrees of freedom and the use of the effective sample size to predict future observations. Additionally, an algebraic proof for the equivalence of these two methods in case of one‐way random effect model is given in the Appendix. An intense simulation study with a wide variety of designs will be made to compare the different intervals and to assess their coverage probabilities. Two real data sets will be used to illustrate and compare the different statistical intervals: one from orthopedic surgery to evaluate the risk of intralesional resection bone tumor (a mixed model with fixed and random variables) and the other from an assay validation study (a one‐way random effect model).

The proposed PI and TI can be useful for a large number of applications in medical research under the linear mixed model framework. The use of PIs for inferring on a future observation deserves more attention from researchers since it quantifies the total uncertainty of measurements taking into account the systematic bias (by using common CI) as well as the precision. The former has been widely accepted and used, while the latter should be equally addressed by researcher for its use when making interpretation of research results. Moreover, the rarely used TI by in‐cooperating the level of confidence would bring additional information to medical research area when a higher level of assurance is needed.

## 2 CONFIDENCE, PREDICTION, AND TOLERANCE FOR A UNIVARIATE MODEL

In this section, we briefly review the classical CI for a mean, the PI for a single future observation and the TI for a proportion of future observations. This will be useful to the understanding on mixed models, to be discussed later. Given that the random variable *X* is normally distributed, it follows *X*∼*N*(*μ*,*σ* ^2^), where *μ* is the mean and *σ* ^2^ the variance, both assumed unknown and estimated, respectively, by $\bar{X}$ and *S* ^2^ (the unbiased estimator of the variance). The classical 100(1−*α*)*%* CI for the mean is then given by

(1) $$\bar{X}\;\pm \;t_{1-\alpha /2,n-1}S/\sqrt{n},$$

where *t*_1−*α*/2,*n*−1_ is the 100(1−*α*/2)*%* quantile of the *t*‐distribution with *n*−1 degrees of freedom.

To predict a future value, *X*_*n*+1_, the uncertainty is composed of two parts: the variability of the estimated mean and the variability of the random variable itself, ie, $\bar{X}∼N(\mu ,\sigma^{2}/n)$ and *X*_*n*+1_∼*N*(*μ*,*σ* ^2^). It follows that $X_{n+1}-\bar{X}∼N(0,\sigma^{2}(1+1/n))$, where the variance *σ* ^2^ has been factorized. Then, the classical 100(1−*ψ*)*%* PI for a future observation is given by

(2) $$\bar{X}\;\pm \;t_{1-\psi /2,n-1}S\sqrt{1+1/n}.$$

The PI for a single future observation, *X*_*n*+1_, is also called *β*‐expectation TI, because a PI will contain, on average, 100(1−*ψ*)*%* (eg, 95%) of the future data.

**Figure 1:**
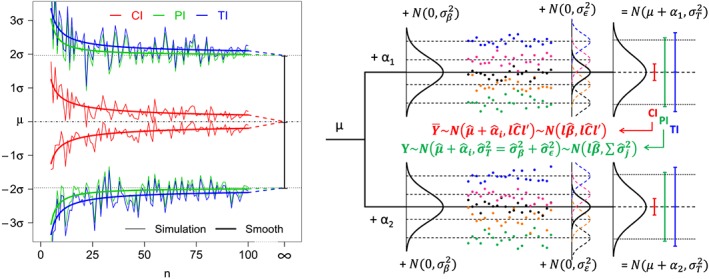
(Left) Comparison of the 1−*α*% confidence interval (CI) for a mean, the 1−*ψ*% prediction interval (PI) for a single future value (nominal levels 95%), and the 1−*ψ*% tolerance interval (TI) with 1−*α*% confidence level (95% prediction with 80% confidence), with respect to the sample size *n*. (Right) Illustration of the CI, PI, and TI for a linear combination of fixed effect in a mixed model with one intercept (*μ*), one fixed variable (*α*: 2 levels) and one random variable (*β*: 5 levels) [Colour figure can be viewed at http://wileyonlinelibrary.com]

The TI type II, also named beta‐gamma content, is an interval in which at least 100(1−*ψ*)*%* of the entire population (future data) will lie with a 100(1−*α*)*%* confidence level. The exact formula is complex and requires to solve an integral, but a good approximate formula can be obtained by replacing the *t* quantile by a *z* quantile (the standardized normal distribution) and the standard deviation by the upper bound of its 100(1−*α*)*%* CI.^11^, ^12^, ^13^ This leads to the following formula:

(3) $$\bar{X}\;\pm \;z_{1-\psi /2}S\sqrt{1+1/n}\sqrt{\frac{n-1}{\chi_{\alpha ,n-1}^{2}}},$$

where *z*_1−*ψ*/2_ is the 100(1−*ψ*/2)*%* quantile of the standardized normal distribution and $\chi_{\alpha ,n-1}^{2}$ is the 100(*α*)*%* quantile of the *χ* ^2^ distribution. A detailed comparison between PI and TI is given in the literature with theoretical aspects and simulation results.^4^ The left panel of Figure 1 summarizes the relationships between these different intervals and their convergence. One sample is simulated for sample size *n*=5 to 100, and the different intervals are calculated by formulas (1), (2), and (3). The lower (upper) bounds are connected for each interval and smoothed curves are added for better visualization. Confidence intervals narrow with increasing sample sizes and collapse to the point estimate (here, the mean *μ*). The PI and TI move closer to the population distribution quantiles, ie, *q*_0.025_ and *q*_0.975_, for 95% PI or 95% TI (whatever its confidence level).

## 3 CONFIDENCE, PREDICTION, AND TOLERANCE IN LINEAR MIXED MODELS

There is no fundamental difference between univariate and mixed models in the context of PI and TI other than the estimation of the parameters and their covariance matrix. The mean of the univariate response will be replaced by the estimate of a given linear combination of fixed effects and the variance by the sum of the variance components in the linear mixed model. The main difficulty lies in estimating the correct degrees of freedom for PI. This section provides briefly the main formulas in estimating a general linear mixed model by the restricted/residual maximum likelihood (REML) method with a variance components covariance structure. More details and other methods can be found in the literature.^14^, ^15^

### 3.1 The linear mixed model and its covariance matrices for the estimated parameters

The linear mixed model is given by the following formula:

(4) Y=Xβ+Zγ+ϵ,

where ***Y*** is the response variable (the known data vector) of length *n*, ***β*** is a vector of *p* unknown parameters corresponding to the fixed effects with known design matrix ***X***, ***γ*** is a vector of *g* unknown parameters corresponding to the random effects with known design matrix ***Z***, and ***ϵ*** is the unknown random error vector. We introduce the notation ***ν*** to define a vector containing both fixed and random effects: ***ν***=(***β***,***γ***). The vectors ***γ*** and ***ϵ*** are assumed to be Gaussian distributed with expectations 0 and their variances are given respectively by ***G*** and ***R*** as follows:

(5) $$\left(\begin{array}{c} \gamma \\ \epsilon \end{array}\right)∼N\left(\left(\begin{array}{c} 0\\ 0 \end{array}\right),\left(\begin{array}{cc} G & 0\\ 0 & R \end{array}\right)\right).$$

The variance of ***Y*** is therefore given by ***V*** as follows:

(6) $$\mathrm{Var}(Y)=V={\mathrm{ZGZ}}^{\prime }+R.$$

The vector of unknown parameters in ***V*** is defined by ***θ***=(*θ*_1_,…,*θ*_*q*_). It includes the residual variance ( $\theta_{q}=\sigma_{\epsilon }^{2}$) and is of length *q*.^16^ The number of unknown parameters in ***θ*** depends on the number of random effects in the model and the residual variance. Different approaches to estimate the parameters in ***G*** and ***R*** are provided in the literature,^16^ but this paper will focus on the REML technique.^17^ If $\hat{V}$ and $\hat{G}$ are the estimates of ***V*** and ***G*** obtained by REML (and $\hat{\theta }$ the estimate of ***θ***), then ***β*** and ***γ*** can be estimated, respectively, by $\hat{\beta }$ and $\hat{\gamma }$ as follows:

(7) $$\hat{\beta }={\left(X^{\prime }{\hat{V}}^{-1}X\right)}^{-}X^{\prime }{\hat{V}}^{-1}Y,$$

(8) $$\;\hat{\gamma }=\hat{G}Z^{\prime }{\hat{V}}^{-1}(Y-X\hat{\beta }),$$

where ^−^ denotes the generalized inverse of a matrix. Note that the variance estimates can be bounded (to zero) to avoid a negative variance component. The approximate variance‐covariance matrix of $\hat{\nu }=(\hat{\beta },\hat{\gamma })$ is then given by $\hat{C}$ 18 as follows:

(9) $$\hat{C}=\left(\begin{array}{cc} {\hat{C}}_{11} & {\hat{C^{\prime }}}_{21}\\ {\hat{C}}_{21} & {\hat{C}}_{22} \end{array}\right)={\left(\begin{array}{cc} X^{\prime }{\hat{R}}^{-1}X & X^{\prime }{\hat{R}}^{-1}Z\\ Z^{\prime }{\hat{R}}^{-1}X & Z^{\prime }{\hat{R}}^{-1}Z+{\hat{G}}^{-1} \end{array}\right)}^{-},$$

where the variance‐covariance matrix of the fixed effects is given by the well‐known formula $\mathrm{Var}(\hat{\beta })={\hat{C}}_{11}={\left(X^{\prime }{\hat{V}}^{-1}X\right)}^{-}$, the covariance between the fixed and random effects is given by ${\hat{C}}_{21}=-\hat{G}Z^{\prime }{\hat{V}}^{-1}X{\hat{C}}_{11}$, and the variance‐covariance matrix of the random effects is given by $\mathrm{Var}(\hat{\gamma })={\hat{C}}_{22}={\left(Z^{\prime }{\hat{R}}^{-1}Z+{\hat{G}}^{-1}\right)}^{-1}-{\hat{C}}_{21}X^{\prime }{\hat{V}}^{-1}Z\hat{G}$. Note that $\hat{\beta }$ and $\hat{\gamma }$ are, respectively, the empirical best linear unbiased estimator and empirical best linear unbiased predictor (EBLUP) as ***G*** and ***R*** are replaced by $\hat{G}$ and $\hat{R}$. Other approximations can be found in the literature, especially useful for unbalanced models, in small sample sizes, or by using the “sandwich” estimator.^19^, ^20^, ^21^, ^22^, ^23^, ^24^ The covariance matrix of maximum likelihood estimates can be estimated by using the Fisher information matrix.^25^ The mathematical details are given by Wolfinger et al.^16^ The covariance matrix of $\hat{\theta }$, here, denoted by $\hat{\vartheta }$, can be estimated by using the (observed) Fisher information matrix (***I***_Obs_), which is the inverse of Hessian matrix:

(10) $$\hat{\vartheta }=I_{\text{Obs}}^{-1}.$$

Finally, $\hat{\beta }$ and $\hat{\theta }$ are asymptotically independent:^26^, ^27^(p239),^28^(p33)

(11) $$\text{Cov}(\hat{\beta },\hat{\theta })\approx 0.$$

Different approximations are available in the literature to calculate the CIs for the fixed effects, but Kenward‐Roger's method for the degrees of freedom, *k*, has been considered.^29^ In SAS, the options OUTPM and OUTP provide the predicted values with the confidence levels for, respectively, a given level of fixed effects or a given level of fixed effects conditioned on random effects:

(12) $$l\hat{\beta }\;\pm \;t_{1-\alpha /2,k}\sqrt{l{\hat{C}}_{11}l^{\prime }},$$

(13) $$l\hat{\nu }\;\pm \;t_{1-\alpha /2,k}\sqrt{l\hat{C}l^{\prime }}.$$

### 3.2 Prediction interval in a linear mixed model

Like the univariate model, the uncertainty to predict a future value in a mixed model, *Y*_*n*+1_, is composed of two parts: the variability of the fixed effects and the total variability (but these two parts cannot be, here, factorized):

(14) Prediction Variance=Fixed effects variance+Total variance.

For a given and estimable linear combination of fixed effects (***lβ*** estimated by $l\hat{\beta }$), the mean, ${\bar{Y}}_{l\beta }$ and the future value, *Y*_*n*+1_, follow respectively these normal distributions:

(15) $$\;{\bar{Y}}_{l\beta }∼N\left(l\beta ,{\mathrm{lC}}_{11}l^{\prime }\right),$$

(16) $$Y_{l\beta ,n+1}∼N\left(l\beta ,\sigma_{T}^{2}\right),$$

where $\sigma_{T}^{2}$ is the “total” variance^30^:

(17) $$\sigma_{T}^{2}=\overset{q}{\underset{i=1}{\sum }}\theta_{i}$$

and estimated by

(18) $${\hat{\sigma }}_{T}^{2}=\overset{q}{\underset{i=1}{\sum }}{\hat{\theta }}_{i}.$$

Formulas (15) and (16) are illustrated on the right panel of Figure 1. As explained in the previous section, a 100(1−*ψ*)*%* PI will converge to the true corresponding quantiles 100(*ψ*/2)*%* and 100(1−*ψ*/2)*%* of distribution (16). These quantiles are here given by

(19) $$l\beta \;\pm \;z_{1-\psi /2,}\sigma_{T}.$$

By combining (15) and (16), it follows that

(20) $$Y_{l\beta ,n+1}-{\bar{Y}}_{l\beta }∼N\left(0,{\mathrm{lC}}_{11}l^{\prime }+\sigma_{T}^{2}\right).$$

A 100(1−*ψ*)*%* PI is then given by

(21) $${Ŷ}_{l\beta ,n+1}\;\pm \;t_{1-\psi /2,r}\sqrt{l{\hat{C}}_{11}l^{\prime }}+{\hat{\sigma }}_{T}^{2},$$

where[Correction added on 20 November 2019 after first online publication: Equation (21) has been corrected.]
${Ŷ}_{l\beta ,n+1}=l\hat{\beta }$. The key parameter in formula (21) is the degrees of freedom *r*. Satterthwaite approximation can be used, but this approach assumes that the variance components are independent.^31^ This assumption may be violated for complex designs, unbalanced designs, or missing data, and also for balanced design, if one is not careful to use the mean‐squares (mean squares are independent) rather than the variance components. The methodology proposed here is therefore based on the generalized Satterthwaite method where the degrees of freedom of a variance can be directly calculated from the variance itself and its standard error (SE) (this equation is more general than the traditional Satterthwaite approximation, as it can even be applied to a linear combination of any distribution, as long as the resulting distribution can reasonably be assumed as chi‐squared):

(22) $$\text{Degrees of Freedom}=2{\left(\frac{\text{Variance}}{\text{SE(Variance)}}\right)}^{2}=2\frac{{\text{Variance}}^{2}}{\text{Var(Variance)}}.$$

The degrees of freedom for the PI in formula (21) is therefore given by

(23) $$r=2\frac{{\hat{\sigma }}_{T}^{4}}{\mathrm{Var}\left({\hat{\sigma }}_{T}^{2}\right)},$$

where $\mathrm{Var}({\hat{\sigma }}_{T}^{2})$ can be replaced by $\hat{\mathrm{Var}}({\hat{\sigma }}_{T}^{2})$ as follows:

(24) $$\hat{\mathrm{Var}}\left({\hat{\sigma }}_{T}^{2}\right)=\overset{q}{\underset{i=1}{\sum }}\overset{q}{\underset{j=1}{\sum }}{\hat{\vartheta }}_{ij},$$

where ${\hat{\vartheta }}_{ij}$ is the (*i*,*j*) element of $\hat{\vartheta }$ (the observed Fisher information matrix).

As a particular case, the PI formula, in the context of a one‐way random balanced design, is usually written as^8^:

(25) $$\hat{\mu }\;\pm \;t_{1-\psi /2,r^{\prime }}{\hat{\sigma }}_{T}\sqrt{1+1/N_{e}},$$

where $\hat{\mu }$ is the estimated intercept (the only fixed effect of the model), *r* *′* is the degree of freedom of the total variance (computed from the mean squares), and *N*_*e*_ is the “effective sample size.” One can notice, after some algebraic manipulations, that our formula (21) is more general and actually includes formula (25). Algebraic proof is given in Appendix A.

Note that each bound of the PI converges to the true quantiles of the normal distribution $N\left(l\beta ,\sigma_{T}^{2}\right)$ (see (19) and Figure 1 (Right)), when the sample size increases (up to infinity) as *t*_1−*ψ*/2,*r*_→*z*_1−*ψ*/2_, ${\hat{C}}_{11}\to 0$, and ${\hat{\sigma }}_{T}^{2}\to \sigma_{T}^{2}$.

To calculate a PI conditionally on a linear combination of fixed and random effects (***lν***), the previous formulas can be adapted where ${\hat{\sigma }}_{T}^{2}$ is replaced by ${\hat{\sigma }}_{\epsilon }^{2}$ (the residual variance is the only one to account for variability) and ${\hat{C}}_{11}$ by $\hat{C}$. This should be done cautiously as the EBLUP are not appropriate for such use, and one should rather consider the random effects as fixed effects, in this purpose, throughout the whole modeling process.

### 3.3 Tolerance interval in a linear mixed model

As explained in Section 2, a PI can also be named *β*‐expectation TI as it contains a given proportion of the future values, but only on average. TIs (type II) are therefore a better approach,^4^ especially for small sample sizes, as a confidence level is added. Sharma and Mathew proposed to calculate two‐sided TIs from small‐sample asymptotics (SSA) approach.^10^ This requires to reparametrize the mixed model and to rewrite the log‐likelihood function.^10^(p262) This methodology is therefore not available from any mixed models output from standard statistical software. Furthermore, the convergence is not achieved when the estimated covariance matrix is not positive definite and the authors propose “to add a small number to the diagonal elements” to tackle this issue.^10^(p263) In this section, we propose an approximate solution where the total variance is replaced by the upper bound of its CI (by analogy to the univariate case). Graybill and Wang^32^ proposed a mathematical method to calculate a CI for a linear combination of variances, called MLS method, which has been later extended for variance components unrestricted in sign.^33^ The TI proposed in this paper is then similar to the PI where the total variance is replaced by the upper bound of its CI obtained by the MLS method on the EMSs (the EMSs are independent in balanced designs, while the variance components may be correlated). The total variance is rewritten as a linear combination of EMSs of each variance components: ${\hat{\sigma }}_{T}^{2}=\sum_{j=1}^{q}k_{j}{\text{EMS}}_{j}$. A 100(1−*ψ*)*%* TI with 100(1−*α*)*%* confidence level is then given by

(26) $${Ŷ}_{l\beta ,n+1}\;\pm \;z_{1-\psi /2}\sqrt{l{\hat{C}}_{11}l^{\prime }+{\hat{\sigma }}_{T}^{2}}\sqrt{1+\frac{1}{{\hat{\sigma }}_{T}^{2}}\sqrt{\overset{q}{\underset{j=1}{\sum }}H_{j}^{2}k_{j}^{2}{\text{EMS}}_{j}^{2}}},$$

where EMS_*j*_ is the EMS associated to the *j*th variance component, and $H_{j}=r_{j}/\chi_{\alpha ,r_{j}}^{2}-1$, where *r*_*j*_ is the classical ANOVA degrees of freedom associated to the corresponding *j* ^*th*^ EMS. Some examples are detailed in the next sections. For other designs or unbalanced cases, the total variance can still be expressed as a linear combination of EMSs, although the MLS method assumes the terms to be independent (simulations and real data example for unbalanced designs are given in the next sections). This TI (26) converges to the PI (21) and to the true quantiles (19) when the sample size (related to the degrees of freedom *r*_*j*_) increases as ${\hat{C}}_{11}\to 0$, ${\hat{\sigma }}_{T}^{2}\to \sigma_{T}^{2}$, and $r_{j}/\chi_{\alpha ,r_{j}}^{2}\to 1$. Note that the MLS method was proposed by Hoffman and Kringle in random effects models,^7^ while the proposed formula (26) is an extension that includes fixed effects of any type. Furthermore, the variance of fixed effects is not taken into account in the MLS approach (26) because calculating the EMSs corresponding to the fixed effects is challenging, especially for unbalanced or complex designs, and the total variance is usually much greater than the variance of fixed effects (as ${\hat{C}}_{11}\to 0$).

#### 3.3.1 Tolerance interval with one random variable

In a mixed model with one random variable and for a given linear combination of fixed effects, we have *Y*_***lβ***,*ij*_=***lβ***+*α*_*i*_+*ϵ*_*ij*_, where *A* is the number of levels of *α*, *n* is the number of replicates, $\alpha_{i}∼N(0,\sigma_{\alpha }^{2})$, and $\epsilon_{ij}∼N(0,\sigma_{\epsilon }^{2})$. The EMS of the residual and the random variable are given, respectively, by $\text{EMSE}={\hat{\sigma }}_{\epsilon }^{2}$ and $\text{EMSA}={\hat{\sigma }}_{\epsilon }^{2}+n{\hat{\sigma }}_{\alpha }^{2}$. It follows that the total variance is given by ${\hat{\sigma }}_{T}^{2}=\text{EMSA}/n+(1-1/n)\text{EMSE}$. The 100(1−*ψ*)*%* TI with 100(1−*α*)*%* confidence level is then given by

(27) $${Ŷ}_{l\beta ,n+1}\;\pm \;z_{1-\psi /2}\sqrt{l{\hat{C}}_{11}l^{\prime }+{\hat{\sigma }}_{T}^{2}}\sqrt{1+\frac{1}{{\hat{\sigma }}_{T}^{2}}\sqrt{H_{A}^{2}{\left(1/n\right)}^{2}{\left(n{\hat{\sigma }}_{\alpha }^{2}+{\hat{\sigma }}_{\epsilon }^{2}\right)}^{2}+H_{\epsilon }^{2}{\left(1-1/n\right)}^{2}{\hat{\sigma }}_{\epsilon }^{4}}},$$

where

(28) $$H_{A}=\frac{A-1}{\chi_{\alpha ,A-1}^{2}}-1\;\text{and}\;H_{\epsilon }=\frac{A(n-1)}{\chi_{\alpha ,A(n-1)}^{2}}-1$$

#### 3.3.2 Tolerance interval with two nested random variables

In a mixed model with two nested random variables and for a given linear combination of fixed effects, we have *Y*_***lβ***,*ijk*_=***lβ***+*α*_*i*_+*β*_*j*(*i*)_+*ϵ*_*j*(*i*)*k*_, where *A* is the number of levels of *α*, *B* is the number of levels of *β*, *n* is the number of replicates, $\alpha_{i}∼N(0,\sigma_{\alpha }^{2})$, $\beta_{j(i)}∼N(0,\sigma_{\beta }^{2})$, and $\epsilon_{j(i)k}∼N(0,\sigma_{\epsilon }^{2})$. The EMS of the residual and the random variables are given, respectively, by $\text{EMSE}={\hat{\sigma }}_{\epsilon }^{2}$, $\text{EMSB}={\hat{\sigma }}_{\epsilon }^{2}+n{\hat{\sigma }}_{\beta }^{2}$ and $\text{EMSA}={\hat{\sigma }}_{\epsilon }^{2}+n{\hat{\sigma }}_{\beta }^{2}+nB{\hat{\sigma }}_{\alpha }^{2}$. It follows that the total variance is given by ${\hat{\sigma }}_{T}^{2}=(1-1/n)\text{EMSE}+\text{EMSA}/(nB)+\text{EMSB}(1/n-1/(nB))$. The 100(1−*ψ*)*%* TI with 100(1−*α*)*%* confidence level is then given by

(29) $${Ŷ}_{l\beta ,n+1}\;\pm \;z_{1-\psi /2}\sqrt{l{\hat{C}}_{11}l^{\prime }+{\hat{\sigma }}_{T}^{2}}\sqrt{1+\frac{1}{{\hat{\sigma }}_{T}^{2}}\sqrt{H_{A}^{2}k_{A}^{2}{\text{EMSA}}^{2}+H_{B}^{2}k_{B}^{2}{\text{EMSB}}^{2}+H_{\epsilon }^{2}k_{\epsilon }^{2}{\hat{\sigma }}_{\epsilon }^{4}}},$$

where

(30) $$\;k_{A}=1/(nB),\;k_{B}=1/n-1/(nB)\;\text{and}\;k_{\epsilon }=1-1/n,$$

(31) $$H_{A}=\frac{A-1}{\chi_{\alpha ,A-1}^{2}}-1,\;H_{B}=\frac{A(B-1)}{\chi_{\alpha ,A(B-1)}^{2}}-1\;\text{and}\;H_{\epsilon }=\frac{AB(n-1)}{\chi_{\alpha ,AB(n-1)}^{2}}-1$$

#### 3.3.3 Tolerance interval with two crossed random variables

In a mixed model with two crossed random variables and for a given linear combination of fixed effects, we have *Y*_*lβ*,*ijk*_=***lβ***+*α*_*i*_+*β*_*j*_+*αβ*_*ij*_+*ϵ*_*ijk*_, where *A* is the number of levels of *α*, *B* is the number of levels of *β*, *n* is the number of replicates, $\alpha_{i}∼N(0,\sigma_{\alpha }^{2})$, $\beta_{j}∼N(0,\sigma_{\beta }^{2})$, $\alpha \beta_{ij}∼N(0,\sigma_{\alpha \beta }^{2})$, and $\epsilon_{ijk}∼N(0,\sigma_{\epsilon }^{2})$. The EMS of the residual, the random variables, and their interaction (EMSAB), are given, respectively, by $\text{EMSE}={\hat{\sigma }}_{\epsilon }^{2}$, $\text{EMSA}={\hat{\sigma }}_{\epsilon }^{2}+n{\hat{\sigma }}_{\alpha \beta }^{2}+nB{\hat{\sigma }}_{\alpha }^{2}$, $\text{EMSB}={\hat{\sigma }}_{\epsilon }^{2}+n{\hat{\sigma }}_{\alpha \beta }^{2}+nA{\hat{\sigma }}_{\beta }^{2}$ and $\text{EMSAB}={\hat{\sigma }}_{\epsilon }^{2}+n{\hat{\sigma }}_{\alpha \beta }^{2}$. It follows that the total variance is given by ${\hat{\sigma }}_{T}^{2}=(1-1/n)\text{EMSE}+\text{EMSA}/(nB)+\text{EMSB}/(nA)+\text{EMSAB}(1/n-1/(nA)-1/(nB))$. The 100(1−*ψ*)*%* TI with 100(1−*α*)*%* confidence level is then given by

(32) $${Ŷ}_{l\beta ,n+1}\;\pm \;z_{1-\psi /2}\sqrt{l{\hat{C}}_{11}l^{\prime }+{\hat{\sigma }}_{T}^{2}}\sqrt{1+\frac{1}{{\hat{\sigma }}_{T}^{2}}\sqrt{H_{A}^{2}k_{A}^{2}{\text{EMSA}}^{2}+H_{B}^{2}k_{B}^{2}{\text{EMSB}}^{2}+H_{AB}^{2}k_{AB}^{2}{\text{EMSAB}}^{2}+H_{\epsilon }^{2}k_{\epsilon }^{2}{\hat{\sigma }}_{\epsilon }^{4}}},$$

where

(33) $$\;k_{A}=1/(nB),\;k_{B}=1/(nA),\;k_{AB}=1/n-1/(nA)-1/(nB)\;\text{and}\;k_{\epsilon }=1-1/n,$$

(34) $$H_{A}=\frac{A-1}{\chi_{\alpha ,A-1}^{2}}-1,\;H_{B}=\frac{B-1}{\chi_{\alpha ,B-1}^{2}}-1,\;H_{AB}=\frac{\left(A-1)(B-1\right)}{\chi_{\alpha ,(A-1)(B-1)}^{2}}-1\;\text{and}\;H_{\epsilon }=\frac{AB(n-1)}{\chi_{\alpha ,AB(n-1)}^{2}}-1$$

### 3.4 Parameterization of fixed and random effects in the linear mixed model

For ease of computation for factor level means and their degrees of freedom, we formulate fixed factor(s) as cell means where no intercept is used and each level of fixed effect combination will be estimated as a parameter in the model directly. The degrees of freedom for the cell means can be approximated using Kenward‐Roger or Satterthwaite method as usual.

For random factor(s) in the model, it is recommended to reflect the actual design of the experiment, where no model simplification is used. Omitting some random effects or combining multiple random factors into one can result in the underestimation of variance components, which is not desirable when predicting a future observation due to the smaller total variance obtained from the model. Moreover, note that building a model to 'explain' or 'understand' the data is not necessarily the same than building a model to 'predict' future values (see the work of Shmueli^34^). The goal is not here to reduce the model or to understand the interaction but to predict future values at a given linear combination of fixed effects.

### 3.5 Statistical software

In SAS, the option ASYCOV from the Proc Mixed procedure provides the covariance matrix of the variance components, ie, the (observed) Fisher information matrix. The options OUTPM and OUTP in the MODEL statement provide the estimated average values for a given level of fixed effects (see (12)) and a given level of fixed effects conditional on random effects (see (13)), respectively. There is no option to calculate PIs for a future observation. The EMSs can be requested from the Proc Mixed statement (ie, TYPE3). In JMP, the “Fit Model” platform allows to run a mixed model and the PIs can be obtained from the “individual CI” option in the “Save Columns” menu. There is no formula and no discussion in the help regarding the PI and its degrees of freedom.^35^ One can actually notice that the degrees of freedom for fixed effects (ie, Kenward‐Roger) are used for the PI. This is not appropriate as explained in Section 3.2 with PIs too wide (see next section). In R, the R package varComp^36^ allows to provide the covariance matrix of the variance components from the function vcov (but varComp is no longer maintained in the current version of R). There is no solution to calculate PIs or TIs, either, other than using a bootstrap approach (ie, from lme4::bootMer). R and SAS code are available in the supplementary material to calculate the CI, PI, and TI presented in this paper and applied in Section 5.

## 4 SIMULATION RESULTS

In order to compare the widths and coverage probabilities of the CIs and our PIs and TIs, 10^4^ data sets were simulated (per scenario) under different scenarios as summarized in Table 1. Three different models are simulated with the intercept as fixed effect, and one random variable, two nested random variables, or two crossed random variables. These models are simulated with a low or high residual variability. Eight different statistical intervals are calculated for each simulated data set: the 95*%* CI (degrees of freedom from Kenward‐Roger's method), the 95*%* PI (degrees of freedom from Kenward‐Roger's method, and from the total variance as proposed in this paper) and the 95*%*TI with confidence levels from 50*%* to 90*%* (step 10*%*). The following sections will summarize the results on graphs with the coverage probabilities, while the comparison of the widths of these intervals is given online as supplementary materials (CI, PI, and the largest TI with 90*%* confidence level). The coverage probabilities are calculated as follows: frequency of simulated CIs that include the true intercept (or the true linear combination of fixed effect). The prediction level of the PIs is assessed by the average of the proportions covered by the simulated PIs (see formula (16)), same for the prediction level of the TIs. The confidence level of the TIs is the frequency of simulated TIs that cover a proportion higher than its prediction level (if the true distribution is an *N*(0,1), and if a 95*%* TI with 80*%* confidence is calculated as [‐2, 2], then it covers a proportion equal to 95.45*%*, and this is higher than 95*%* as it should be in 80*%* of cases).

**Table 1:** Six different models used to simulate the mixed models: 1 random variable, 2 random factors (1 factor nested), and 2 random factors crossed, with high or low residual variance

Variance components (% of the total variability)						
	Higher residual variance			Lower residual variance		
	One	Two nested	Two crossed	One	Two nested	Two crossed
$\sigma_{\alpha }^{2}$	2 (20%)	2 (20% )	1 (10%)	8 (80%)	5 (50%)	4 (40%)
$\sigma_{\beta }^{2}$			1 (10%)			2 (20%)
$\sigma_{\alpha \beta }^{2}$		2 (20%)	2 (20%)		3 (30%)	2 (20%)
$\sigma_{\epsilon }^{2}$	8 (80%)	6 (60%)	6 (60%)	2 (20%)	2 (20%)	2 (20%)
$\sigma_{T}^{2}$ (Total)	10 (100%)	10 (100%)	10 (100%)	10 (100%)	10 (100%)	10 (100%)

### 4.1 One random variable

**Figure 2:**
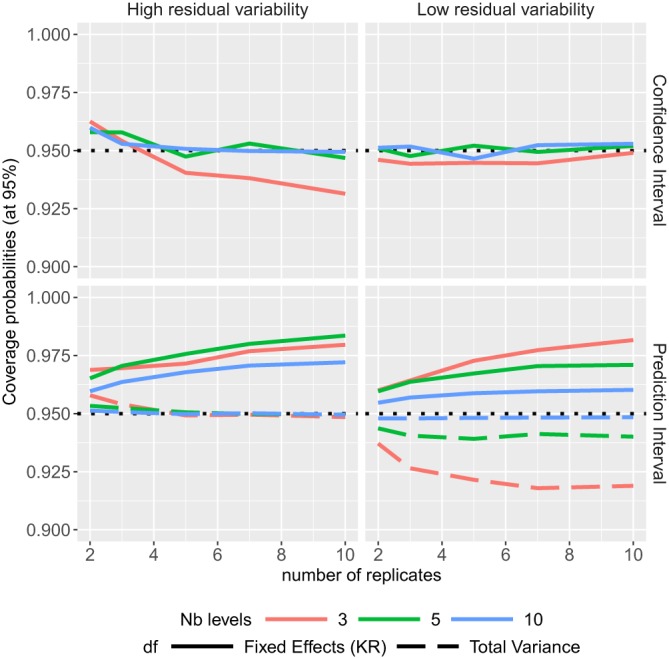
Coverage probabilities of the confidence and prediction intervals in one random variable model, according to different sample sizes [Colour figure can be viewed at http://wileyonlinelibrary.com]

**Figure 3:**
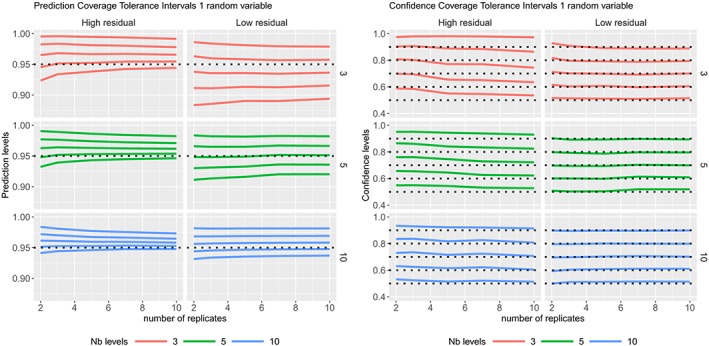
Coverage probabilities of the 95% tolerance intervals with 50%, 60%, 70%, 80%, and 90% (5 curves from bottom to top) confidence levels, in one random variable model with high or low residual variability, according to different sample sizes. Prediction levels are on the left side, and confidence levels are on the right. KR, Kenward‐Roger's method [Colour figure can be viewed at http://wileyonlinelibrary.com]

The model used for the simulations is *Y*_*ij*_=*μ*+*α*_*i*_+*ϵ*_*ij*_, where *i*, the number of levels of *α*, is equal to 3, 5, or 10 and *j*, the number of replicates, is equal to 2, 3, 5, 7 or 10, which leads to 15 different sample sizes. Furthermore, the intercept *μ*=25, the random effects $\alpha_{i}∼N(0,\sigma_{\alpha }^{2})$, and the residual $\epsilon_{ij}∼N(0,\sigma_{\epsilon }^{2})$ where the variance components values are given in Table 1. The coverage probabilities of the CIs and PIs are summarized in Figure 2 and the TIs in Figure 3.

The coverage probabilities of the CIs of factor level means obtained by Kenward‐Roger's method are very good, except for a low number of levels of the random variable when the residual variability is high and the number of replicates is high (see Figure 2). The coverage probabilities of the PIs, with degrees of freedom calculated form the total variance, are close to the nominal level except that when the residual variability is low, and there is a low number of levels in the random variable. Note that three levels for a random variable is really a minimum, and the coverage probabilities are higher than 93% from 5 levels. The PIs calculated with degrees of freedom from Kenward‐Roger's method are conservative as their coverage probabilities are always higher than the nominal level. Most of the prediction levels of the TIs are higher than the nominal level (see Figure 3 (Left)), as expected, because these intervals include at least 95*%* of the future data. The higher the confidence level, the wider the TIs, and on Figure 3 (Right), we can notice that the confidence levels are close to their nominal levels, except when the residual variability is high with a low number of levels for the random variable. The average widths of TIs (with 90*%* confidence level) are larger than those of PIs, which are larger than those of CIs (see supplementary materials), as expected, whatever the design (sample size). Furthermore, none of the simulated PIs were narrower than the simulated CIs for each simulated data set. While this comment may seem surprising as this is not expected, the comment is noteworthy as in later simulations for specific parameter combinations this seemingly paradoxical behavior can be observed and will be further discussed.

### 4.2 Two nested random variables

**Figure 4:**
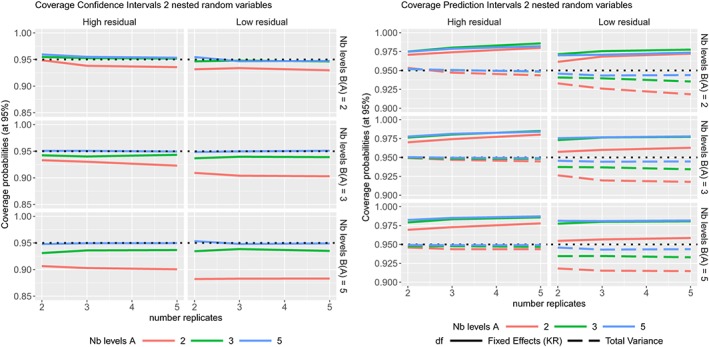
Coverage probabilities of the 95% confidence intervals (Left) and 95% prediction intervals (Right) in two nested random variables model, according to different sample sizes [Colour figure can be viewed at http://wileyonlinelibrary.com]

**Figure 5:**
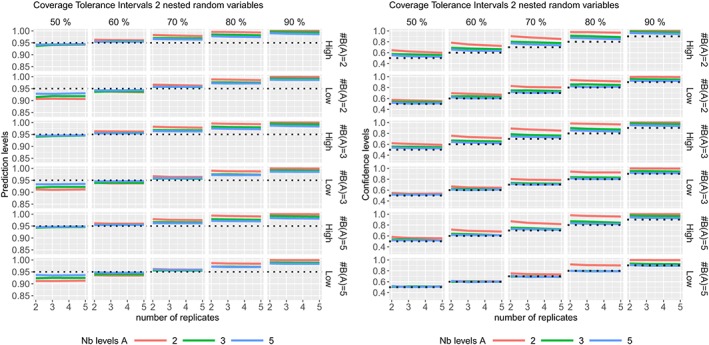
Coverage probabilities of the prediction levels (at nominal level 95%) (Left) and confidence levels (Right) of the tolerance intervals (with 50%, 60%, 70%, 80%, and 90% confidence levels) in two nested random variables model with high or low residual variability, according to different sample sizes [Colour figure can be viewed at http://wileyonlinelibrary.com]

The model used for simulation is *Y*_*ijk*_=*μ*+*α*_*i*_+*β*_*j*(*i*)_+*ϵ*_*j*(*i*)*k*_, where *i* the number of levels of *α* is equal to 2, 3, or 5; *j* the number of levels of *β* within *α* is equal to 2, 3, or 5; and *k* the number of replicates is equal to 2, 3, or 5, which leads to 27 different sample sizes. Furthermore, the intercept *μ*=25, the random effects $\alpha_{i}∼N(0,\sigma_{\alpha }^{2})$, $\beta_{j(i)}∼N(0,\sigma_{\beta }^{2})$, and the residual $\epsilon_{j(i)k}∼N(0,\sigma_{\epsilon }^{2})$ where the variance components values are given in Table 1. The coverage probabilities of the CIs and PIs are summarized in Figure 4, the TIs in Figure 5, while their average widths are summarized in the supplementary materials.

The coverage probabilities of the CIs obtained by Kenward‐Roger's method are very good, except for a low number of levels of the random variable (Figure 4 (Left)). The coverage probabilities of the PIs, with degrees of freedom calculated form the total variance, are excellent except when the residual variability is low with a low number of levels of the random variable (as it is for the one random variable model). The PIs calculated with degrees of freedom from Kenward‐Roger's method are conservative as their coverage probabilities are always higher than the nominal level. Most of the prediction levels of the TIs are higher than the nominal level (see Figure 5) for a high residual variability as expected. In theory, a TI with 50% confidence is similar to a PI (the average is nearly equal to the median), but in practice, a 60% confidence level is usually required.^4^ This is also observed here in Figure 5 where the TIs with 60% confidence level are very close to the 95% prediction level. On Figure 5, the confidence levels are close to their nominal levels except for very low number of levels from the random variable (ie, two levels).

### 4.3 Two crossed random variables

**Figure 6:**
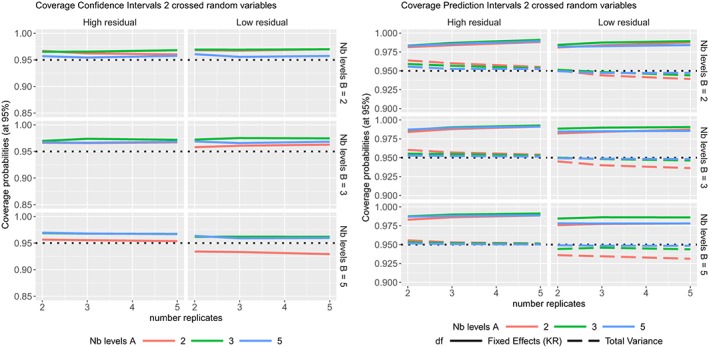
Coverage probabilities of the (Left) 95% confidence intervals and (Right) 95% prediction intervals in two crossed random variables model, according to different sample sizes [Colour figure can be viewed at http://wileyonlinelibrary.com]

**Figure 7:**
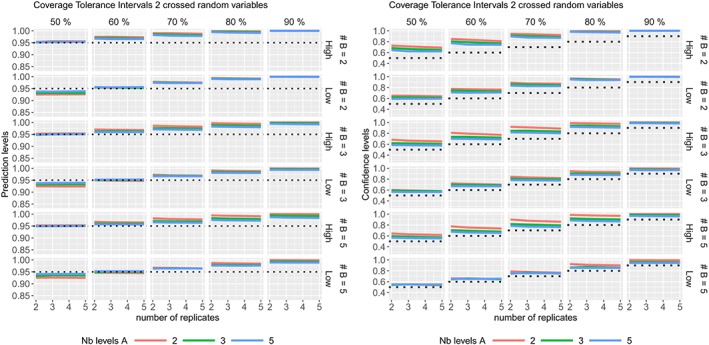
Coverage probabilities of the (Left) prediction levels (at nominal level 95%) and (Right) confidence levels of the tolerance intervals (with 50%, 60%, 70%, 80%, and 90% confidence levels) in two crossed random variables model with high or low residual variability, according to different sample sizes [Colour figure can be viewed at http://wileyonlinelibrary.com]

The model used for simulation is *Y*_*ijk*_=*μ*+*α*_*i*_+*β*_*j*_+*αβ*_*ij*_+*ϵ*_*ijk*_, where *i* the number of levels of *α* is equal to 2, 3, or 5, *j* the number of levels of *β* is equal to 2, 3, or 5, and *k* the number of replicates is equal to 2, 3, or 5 ,which leads to 27 different sample sizes. Furthermore, the intercept *μ*=25, the random effects $\alpha_{i}∼N(0,\sigma_{\alpha }^{2})$, $\beta_{j}∼N(0,\sigma_{\beta }^{2})$, $\alpha \beta_{ij}∼N(0,\sigma_{\alpha \beta }^{2})$, and the residual $\epsilon_{ijk}∼N(0,\sigma_{\epsilon }^{2})$ where the variance components values are given in Table 1. The coverage probabilities of the CIs and PIs are summarized in Figures 6, the TIs in Figure 7, while their average widths are summarized in the supplementary materials.

Most of the coverage probabilities of the CIs obtained by Kenward‐Roger's method are slightly higher than the nominal level, Figure 6 (Left). The coverage probabilities of the PIs, with degrees of freedom calculated form the total variance, are excellent except when the residual variability is low with a low number of levels of the random variable (see Figure 6 (Right)). The PIs calculated with degrees of freedom from Kenward‐Roger's method are conservative as their coverage probabilities are always higher than the nominal level. Most of the prediction levels of the TIs are higher than the nominal level (see Figure 7 (Left)) as expected. The TIs with 60% confidence level are very close to the 95% prediction level, as expected. On Figure 7 (Right), the coverage probabilities are higher than their nominal levels when the residual variability is high. The coverage probabilities move closer to the nominal level when the residual variability is low and when the random variables have at least three levels.

### 4.4 Unbalanced two crossed random variables

The simulation scenarios from Sharma and Mathew^10^(see Section 5.2) are used to compare the performance of our methodology (based on the MLS approach) to the SSA approach on an unbalanced two crossed random variables design. The model is similar to the one detailed in the previous section where, here, *α* and *β* have, respectively, four and five levels. The design is largely unbalanced with the number of replicates from 1 to 9 and given by ((1,2,1,9)*′*,(2,9,8,1)*′*,(1,1,1,3)*′*,(7,1,6,2)*′*,(1,3,1,1)*′*). Twelve scenarios are simulated by setting the variance components as follows: $\sigma_{\alpha }^{2}=10,10,10,10,5,5,5,5,1,1,1,1$; $\sigma_{\beta }^{2}=0.1,0.1,0.5,0.5,0.1,0.1,0.5,0.5,0.1,0.1,0.5,0.5$ and $\sigma_{\alpha \beta }^{2}=0.1,0.5,0.1,0.5,0.1,0.5,0.1,0.5,0.1,0.5,0.1,0.5$. The intercept is equal to 0, and the residual variance is 1 for all scenarios. Formulas given in Section 3.3 can still be used as an approximate solution, by adapting the EMS. The EMS of the residual, the random variables and their interaction, are given, respectively, by $\text{EMSE}={\hat{\sigma }}_{\epsilon }^{2}$, $\text{EMSA}={\hat{\sigma }}_{\epsilon }^{2}+1.568{\hat{\sigma }}_{\alpha \beta }^{2}+7.84{\hat{\sigma }}_{\alpha }^{2}$, $\text{EMSB}={\hat{\sigma }}_{\epsilon }^{2}+1.6552{\hat{\sigma }}_{\alpha \beta }^{2}+6.6206{\hat{\sigma }}_{\beta }^{2}$, and $\text{EMSAB}={\hat{\sigma }}_{\epsilon }^{2}+2.0754{\hat{\sigma }}_{\alpha \beta }^{2}$. It follows that the total variance is given by ${\hat{\sigma }}_{T}^{2}=0.464\text{EMSE}+0.128\text{EMSA}+0.151\text{EMSB}+0.265\text{EMSAB}$. Only the 90% TIs with 95% confidence level were simulated by Sharma and Mathew using the small‐sample asymptotic methodology.^10^ We reproduce here their simulations together with the 95% CIs (degrees of freedom by Kenward‐Roger's method) and the 95% PIs with degrees of freedom from the fixed effects (Kenward‐Roger) or from our methodology by using the total variance. The 90*%* TI with 95*%* confidence level is here given by

$$\hat{\mu }\;\pm \;1.645\sqrt{l{\hat{C}}_{11}l^{\prime }+{\hat{\sigma }}_{T}^{2}}\sqrt{1+\frac{1}{{\hat{\sigma }}_{T}^{2}}\sqrt{H_{A}^{2}k_{A}^{2}{\text{EMSA}}^{2}+H_{B}^{2}k_{B}^{2}{\text{EMSB}}^{2}+H_{AB}^{2}k_{AB}^{2}{\text{EMSAB}}^{2}+H_{\epsilon }^{2}k_{\epsilon }^{2}{\hat{\sigma }}_{\epsilon }^{4}}},$$

where

$$\;k_{A}=0.128,\;k_{B}=0.151,\;k_{AB}=0.265\;\text{and}\;k_{\epsilon }=0.464,$$

$$\mathrm{var}\hat{\mu }=l{\hat{C}}_{11}l^{\prime },\;H_{A}=\frac{3}{\chi_{0.05,3}^{2}}-1,\;H_{B}=\frac{4}{\chi_{0.05,4}^{2}}-1,\;H_{AB}=\frac{12}{\chi_{0.05,12}^{2}}-1\;\text{and}\;H_{\epsilon }=\frac{41}{\chi_{0.05,41}^{2}}-1.$$

**Figure 8:**
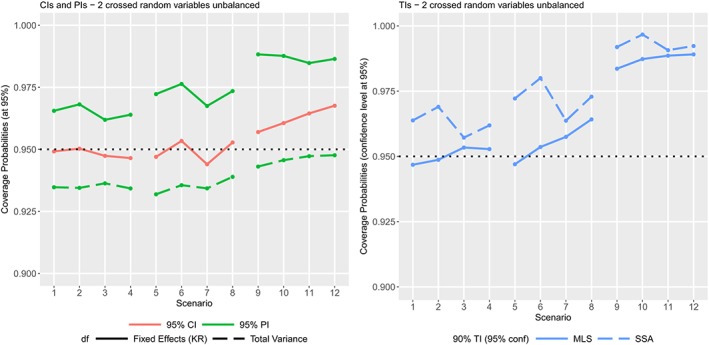
Coverage probabilities of the different statistical intervals for two crossed random variables in 12 unbalanced designs. (Left) 95% confidence interval (CI) and 95% prediction interval (PI) (with degrees of freedom from fixed effects by Kenward‐Roger (KR) or from the total variance). (Right) 90% tolerance intervals (TIs) with 95% confidence levels (from the small‐sample asymptotics (SSA) or modified large sample (MLS) methods) [Colour figure can be viewed at http://wileyonlinelibrary.com]

The coverage probabilities of the CIs obtained by Kenward‐Roger's method are excellent except an overestimation for high residual variability (CIs slightly too wide for the 4 last scenarios on Figure 8 ‐ left). The coverage probabilities of the PIs with degrees of freedom from Kenward‐Roger's method are all higher than the nominal level (the PIs are too wide). The coverage probabilities of the PIs with degrees of freedom from our methodology based on the total variance are better and closer to the nominal level (Figure 8 ‐ left). The coverage probabilities of the TIs calculated with our MLS approach are better than the SSA approach for the first half of designs, and similar for the second half of designs where the coverage probabilities are higher than the nominal level. As explained in the previous sections, the TIs may be conservative when the residual variability is high.

### 4.5 A paradox with confidence interval larger than prediction interval

In the supplementary material, the average widths of TIs are larger than those of PIs, which are larger than those of CIs, as expected, except for the designs with a low number of levels for a random variable (ie, only two levels). In these cases, the 95*%* CIs may be larger than 95*%* PIs, which leads to a paradox as a PI should be larger than a CI. The variance of prediction is always larger than the variance of the fixed effects (see (14)). The degrees of freedom (calculated from the total variance) is also larger (or equal) for a prediction compared to a mean (fixed effects); thus, the quantile of the *t*‐distribution is smaller for a prediction. A paradox arises when the increase of the variance for the prediction does not compensate the decrease of the *t*‐distribution quantile. This paradox appeared in our simulations with nested design only for the design with two levels nested within the parent variable, with a frequency approximately between 40*%* and 70*%* (depending on the number of levels for the parent variable and the residual variability) (see the supplementary online material for plots of frequency). It occurred in crossed design with only two levels for the first or second random variable, with a frequency approximately between 25*%* and 75*%* (depending on the number of levels of the other random variable and the residual variability).

### 4.6 Other designs

The simulations presented in this paper are based on random models with intercept as fixed effect. The results and findings remain valid with additional fixed effects. In case of missing data (under the assumption of missing completely at random or missing at random), the approach proposed remain valid as our calculation is based on the Hessian matrix in the linear mixed model. Note that with missing data, the design is unbalanced, and the variance components may be (highly) correlated. Our approach that the degrees of freedom are calculated directly by using the Generalized Satterthwaite's method takes into consideration the covariance of random effects estimated from the Hessian matrix.

## 5 APPLICATIONS AND DISCUSSION

### 5.1 Orthopedic surgery study, intralesional resection risk ‐ mixed model

The data set from Francq and Cartiaux is used as an illustration to compare the different statistical intervals (see the literature for descriptive plots and more statistical details,^30^ and for more details on the study design and medical context.^37^, ^38^, ^39^). The data and R or SAS code are provided in the Supplementary online material. This study is composed of 23 surgeons performing a tumor cutting at four different slices (fixed variable with four levels) with a free hand or navigated technology (fixed variable with two levels). The response variable is the Error of Safe Margin (ESM) that should ideally be between ‐5 and 5. Values lower than ‐10 mean that the tumor is not removed completely (intralesional resection). The goal of this study was to assess and compare the risk of intralesional resection in bone tumor surgery between the classical surgery (free hand procedure) and a new surgery (navigated technology). The data are assumed to be Gaussian. To simplify the calculation of the predictions as explained in Section 3.4, the model only includes cell means (combinatory factor levels of slice and technology) for the fixed effects, and surgeon as a random variable. The four measurements from the same surgeon are correlated with a different correlation for each technology. The mixed model is estimated by REML technique, with degrees of freedom for fixed effects by Kenward‐Roger's method and covariance matrix of variance components given by the observed Fisher information matrix. The variance components are estimated per technology to tackle the heteroscedasticity by allowing heterogeneity on the covariance structure (the navigated technology provides much lower variability than the free hand). The variance for the random effect of surgeon is estimated to be 7.349 and 0.447, and the residual ones are obtained as 18.074 and 3.611, for respectively the free hand and navigated technology. The total variances are then, respectively, 25.423 and 4.058. The variance of the total variance is given by summing all elements of their estimated covariance matrix, respectively, 18.372 and 0.388. This leads to 70.36 and 84.93 degrees of freedom (calculated from the estimated total variance, see formula (23)). The predicted ESM for slice 1 with free hand technology is 0.114 with a SE equal to 1.051 and 70.4 degrees of freedom. The variance of the prediction is then 1.051^2^+25.423=26.528. For the free hand technology and slice 1, the 95% PIs and TI (with 80% confidence, from MLS method) are then given by

$$95\%PI:0.114\;\pm \;t_{0.975,70.36}\sqrt{26.528}$$

$$95\%TI:0.114\;\pm \;z_{0.975}\sqrt{26.528}\sqrt{1+\frac{1}{25.423}\sqrt{H_{Sur}^{2}{\left(\frac{1}{4}\right)}^{2}{\left(4\cdot 7.349+18.074\right)}^{2}+H_{Res}^{2}{\left(1-\frac{1}{4}\right)}^{2}18.074^{2})}},$$

where the degrees of freedom for surgeon variance is 22 and the residual one is 66 as (23−1)(4−1)=66 (which is slightly different from Equation (28)), $H_{Sur}=22/\chi_{0.2,22}^{2}-1$ and $H_{res}=66/\chi_{0.2,66}^{2}-1$.

**Figure 9:**
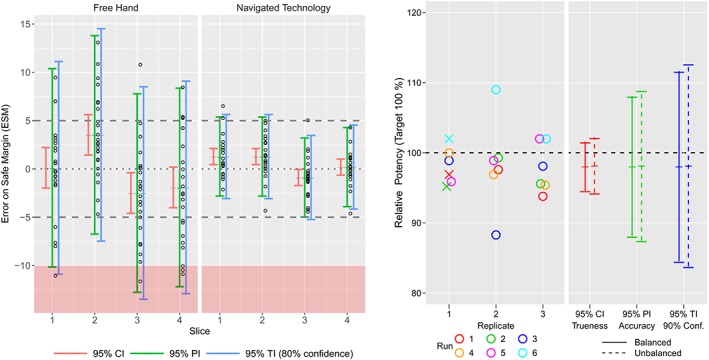
(Left) Orthopedic surgery study data with the 95*%* confidence, prediction, and tolerance intervals (with 80*%* confidence level) on the error of safe margin for free hand technology and navigated technology at the four slices. (Right) Assay validation study data with the 95*%* confidence, prediction, and tolerance intervals (with 90*%* confidence level), balanced or unbalanced design (three removed data marked by a cross) (Right) [Colour figure can be viewed at http://wileyonlinelibrary.com]

The different statistical intervals are displayed in Figure 9 (Left) for free hand and navigated technology at the four slices. Table 2 provides the estimates of cell means and all the estimated values of the three intervals. As expected, the widths of 95% TIs (with 80% confidence) are larger than those of the 95% PIs, which are larger than those of the 95% Cis. If we focus on slice 1 with free hand technology, we predict ESM to be 0.11; the average ESM will be between ‐1.98 and 2.21 (with 95% confidence), whereas 95% of individual ESM values are expected to lie between ‐10.16 and 10.39 on average, or between ‐10.89 and 11.1 with 80*%* confidence (at least 95% of individual ESM values will be between these two values in 80% of cases). Surgeons can fail to remove the tumor with free hand technology at slice 1, 3, and 4 as the PIs or TIs overlap the threshold ‐10.

**Table 2:** Predicted mean values with lower and upper limits of the 95*%* confidence interval (CI) (degrees of freedom from Kenward‐Roger's method), prediction interval (PI) (degrees of freedom from the estimated total variance), and tolerance interval (TI) (with 80*%* confidence level) on error of safe margin (ESM) data at four different slices and free hand (1) or navigated (2) technology

		Predicted		95% CI			95% PI		95% TI (80% conf.)	
Slice	Technology	ESM	DF	Lower	Upper	DF	Lower	Upper	Lower	Upper
1	1	‐0.11	70.4	‐1.98	‐2.21	70.36	‐10.16	10.39	‐10.89	11.11
1	2	‐1.29	84.9	‐0.45	‐2.12	84.93	‐2.80	15.38	‐3.06	15.64
2	1	‐3.53	70.4	‐1.43	‐5.63	70.36	‐6.74	13.80	‐7.47	14.53
2	2	‐1.28	84.9	‐0.45	‐2.12	84.93	‐2.81	15.37	‐3.07	15.63
3	1	‐2.49	70.4	‐4.59	‐0.40	70.36	‐12.77	17.78	‐13.50	18.51
3	2	‐0.89	84.9	‐1.72	‐0.05	84.93	‐4.98	13.20	‐5.24	13.46
4	1	‐1.91	70.4	‐4.01	‐0.18	70.36	‐12.19	8.36	‐12.92	19.09
4	2	‐0.19	84.9	‐0.64	‐1.03	84.93	‐3.90	14.28	‐4.16	14.54

### 5.2 Assay validation study ‐ one‐way random model

During development of a vaccine, different analytical methods for determining the antigen concentration or the (relative) potency in the produced vaccine batches need to be developed. In assay development, a linear mixed model across all samples is applied to estimate the variance components to evaluate the precision of the method (repeatability and intermediate precision). Trueness is the systematic deviation (bias) between the reference value and the measured concentration. Accuracy is the closeness between the reference value and an individual test value, which takes into account the systematic error (trueness) and random error (precision). Trueness is therefore assessed by using CIs at each measured concentration while accuracy is evaluated by using PIs. The aim of a validation is to show that an analytical method is suitable for its intended use, and consequently to prove the reliability of the measurements.

The dataset from Hoffman and Kringle^7^ is used to illustrate the different statistical intervals on a one‐way random model. Concentrations are measured for six runs and three replicates (balanced). The REML method is used, together with Kenward‐Roger's method for degrees of freedom of intercept. The run and residual variances are 0.000681 and 0.001253, their variances are, respectively, 5.123×10^7^ and 2.617×10^7^ (obtained from the observed Fisher information matrix), and their covariance is estimated to −8.72×10^8^. The total variances is then 0.000681+0.001253=0.001934, and its variance 5.123×10^7^+2.617×10^7^+2(−8.72×10^8^)=5.995×10^7^. This leads to 2(0.001934^2^/(5.995×10^07^))=12.484 degrees of freedom (calculated from the estimated total variance and its variance) for the PI. The predicted concentration (intercept) is then 0.981 (with SE = 0.01353) with a 95% CI [0.946,1.016], 95% PI [0.881,1.081], 95% TI (with 90% confidence and MLS method) [0.845,1.117] (only the TI was given by Hoffman and Kringle as [0.846,1.114]). The different statistical intervals are displayed in Figure 9 (Right).

### 5.3 Assay validation study ‐ unbalanced one‐way random model

The same data set^7^ is used to illustrate the different statistical intervals on an unbalanced one‐way random model, where the first replicates of run 1, run 2, and run 6 are taken out (three missing values). The run and residual variances are then 0.000575 and 0.001594; their variances are, respectively, 8.605×10^7^ and 6.151×10^7^ (obtained from the observed Fisher Information matrix), and their covariance is estimated to −3.22×10^7^. The total variances is then 0.000575+0.001594=0.002169 and its variance 8.605×10^7^+6.151×10^7^+2(−3.22×10^7^)=8.316×010^7^. This leads to 2(0.002169^2^/(8.316×10^7^))=11.31448 degrees of freedom for the PI. Formulas (27) and (28) have to be adapted to the unbalanced design by modifying the degrees of freedom and the EMS. The EMS of the residual and the random variable are given here, respectively, by $\text{EMSE}={\hat{\sigma }}_{\epsilon }^{2}$ and $\text{EMSA}={\hat{\sigma }}_{\epsilon }^{2}+2.48{\hat{\sigma }}_{\alpha }^{2}$. It follows that the total variance is given by ${\hat{\sigma }}_{T}^{2}=\text{EMSA}/2.48+(1-1/2.48)\text{EMSE}$. The 95*%* TI with 90*%* confidence is then given by

$$\hat{\mu }\;\pm \;1.96\sqrt{\mathrm{Var}(\hat{\mu })+{\hat{\sigma }}_{T}^{2}}\sqrt{1+\frac{1}{{\hat{\sigma }}_{T}^{2}}\sqrt{H_{A}^{2}{\left(1/2.48\right)}^{2}{\left(2.48{\hat{\sigma }}_{\alpha }^{2}+{\hat{\sigma }}_{\epsilon }^{2}\right)}^{2}+H_{\epsilon }^{2}{\left(1-1/2.48\right)}^{2}{\hat{\sigma }}_{\epsilon }^{4}}},$$

where

$$H_{A}=\frac{5}{\chi_{0.1,5}^{2}}-1\;\text{and}\;H_{\epsilon }=\frac{9}{\chi_{0.1,9}^{2}}-1.$$

The predicted concentration ( $\hat{\mu }$, the intercept) is then 0.9822 (with SE = 0.01441) with a 95% CI [0.943,1.022], 95% PI [0.875,1.089], 95% TI (with 90% confidence) [0.838,1.127]. Note that these statistical intervals are (slightly) larger compared to the balanced case as three values are missing (see Figure 9), the variances are therefore greater and the degrees of freedom smaller.

### 5.4 Discussion

The performance of the predictive analysis (ie, PIs or TIs) can be assessed from a cross‐validation technique or by using a test data set. In both cases, observations that do not contribute to the computation of the statistical intervals are considered as ‘new’ observations and can then be compared to the statistical intervals. Empirical coverage can be obtained, ie, the frequency of ‘new’ observations included in the PIs, and the predictive or confidence level can be adjusted if needed (see also the bootstrap calibration proposed by Hoffman for one‐sided TIs^40^ or the double calibration bootstrap in mixed models proposed by Francq and Cartiaux^30^). Note that these techniques are efficient for large data set while small or moderate sample sizes are usually encountered in non‐clinical or preclinical study (ie, Section 5.2).

Multivariate tolerance regions can be used when multiple attributes are assessed.^3^ In the orthopedic surgery study (Section 5.1>), the primary outcome is the error on safe margin but other responses variables (ie, location, parallelism, etc)^37^, ^38^, ^39^ were measured, and multivariate tolerance regions can be useful to assess these different outcomes simultaneously (ie, the joint TIs where the different outcomes of a future surgery will lie). In assay validation study (Section 5.2) or when measuring vaccines quality, the main response variable is usually the potency but other attributes may also be measured (ie, viral vector aggregation, free viral proteins, polysorbate content, sucrose content, etc) and multivariate tolerance regions can be very helpful to compare new batches to standard or reference samples.

## 6 PRACTICAL RECOMMENDATIONS AND CONCLUSIONS

In this paper, we recommend the use of formula (21) and formula (26) to obtain a two‐sided PI for a future observation without and with an additional confidence level in linear mixed models. The approach of calculating the PI has been shown to be generalizable for more complex designs with its connection to classical Satterthwaite method for the balanced design with one random factor. The MLS approach for TIs provides better accuracy than the small‐sample asymptotic method for low residual variability (similar accuracy otherwise), and is mostly easier to apply in practice. Our simulation study shows good coverage probabilities for the proposed PIs and TIs. Study designs with a small number of levels for a random factor (less than three) are not recommended to be used given that the coverage probability of PI/TI can be lower/higher than their nominal level.

When obtaining the PIs/TIs for a future observation at a given level of fixed effect(s), we recommend building a cell mean model without the intercept, which has the advantage of obtaining degrees of freedom and SE of fixed effect(s) directly from the model. Our solution to the PI and TI can be constructed by using the output of the mixed model from standard software (eg, SAS proc mixed and R lme4 or varComp package).

The proposed analytical solution of PIs and TIs in this paper can be useful and important for medical research and a large number of applications under the linear mixed model framework. It is straightforward to use as compared to other resampling‐based methods and Bayesian method which can be computational intensive and relying on the input parameters (eg, prior distribution of parameters). For further research, we shall compare the results of our proposed method to that of Bayesian TIs using simulation study as well as real case studies.

## Supporting information

SIM_8386‐Supp‐0001‐PI_MixedModels_Supp.pdf

## PROOF EQUALITY OF PREDICTION INTERVALS IN ONE RANDOM VARIABLE MODELS

The PI in the context of a one‐way random balanced design (with intercept *μ*, residual variance $\sigma_{\epsilon }^{2}$, mean square of errors (MSE), variance of the random variable $\sigma_{A}^{2}$, mean squares of the random variable (MSA), number of levels of the random variable *A*, and number of replicates *n*) is usually written as^8^:

$$\hat{\mu }\;\pm \;t_{1-\alpha /2,r^{\prime }}{\hat{\sigma }}_{T}\sqrt{1+1/N_{e}},$$

which is a particular case of our formula (21), as $N_{e}={\hat{\sigma }}_{T}^{2}/{\hat{\sigma }}_{\mu }^{2}$:

$$\hat{\mu }\;\pm \;t_{1-\alpha /2,r^{\prime }}{\hat{\sigma }}_{T}\sqrt{1+{\hat{\sigma }}_{\mu }^{2}/{\hat{\sigma }}_{T}^{2}},$$

$$\;\hat{\mu }\;\pm \;t_{1-\alpha /2,r^{\prime }}\sqrt{\hat{\sigma_{T}^{2}}+{\hat{\sigma }}_{\mu }^{2}}.$$

The only difference with our formula (21) is the parameter *r* *′*, the degrees of freedom. This parameter *r* *′* is calculated from the classical Satterthwaite approximation on a linear combination of mean squares:

$$r^{\prime }=\frac{\left(\sum_{j=1}^{p}c_{j}MS_{j}\right)}{\sum_{j=1}^{p}\frac{c_{j}^{2}MS_{j}^{2}}{r_{j}}},$$

where *r*_*j*_ are the degrees of freedom (of the component *j*) obtained from the classical ANOVA table. The mean squares are used as they are independent (for balanced designs) while the variance components, ${\hat{\sigma }}_{\epsilon }^{2}$ and ${\hat{\sigma }}_{A}^{2}$, are (slightly) correlated. As $\sigma_{\epsilon }^{2}=MSE$ and $\sigma_{A}^{2}=(MSA-MSE)/n$,^27^ ^(p27,92)^ it follows that the total variance $\sigma_{T}^{2}=\sigma_{\epsilon }^{2}+\sigma_{A}^{2}$ can be rewritten as $\sigma_{T}^{2}=\text{MSA}/n+(1-1/n)\text{MSE}$. It follows:

(A1) $$r^{\prime }=\frac{{\left(\left(1/n\right)\text{MSA}+\left(1-1/n\right)\text{MSE}\right)}^{2}}{\frac{{\left(1/n\right)}^{2}{\text{MSA}}^{2}}{A-1}+\frac{{\left(1-1/n\right)}^{2}{\text{MSE}}^{2}}{A(n-1)}}.$$

On the other hand, the degrees of freedom, *r*, for our PI in formula (21) is given by

(A2) $$r=2\frac{{\hat{\sigma }}_{T}^{4}}{\mathrm{Var}\left({\hat{\sigma }}_{T}^{2}\right)}=2\frac{{\left({\hat{\sigma }}_{A}^{2}+{\hat{\sigma }}_{\epsilon }^{2}\right)}^{2}}{\mathrm{Var}\left({\hat{\sigma }}_{T}^{2}\right)}=2\frac{{\left(\left(1/n\right)\text{MSA}+\left(1-1/n\right)\text{MSE}\right)}^{2}}{\mathrm{Var}\left({\hat{\sigma }}_{A}^{2}\right)+\mathrm{Var}\left({\hat{\sigma }}_{\epsilon }^{2}\right)+2\text{Cov}\left({\hat{\sigma }}_{A}^{2},{\hat{\sigma }}_{\epsilon }^{2}\right)}.$$

The covariance matrix between the two variance components is given by^27^ ^(p90)^:

(A3) $$\text{Cov}\left({\hat{\sigma }}_{\epsilon }^{2},{\hat{\sigma }}_{A}^{2}\right)=2{\hat{\sigma }}_{\epsilon }^{4}\left(\begin{array}{cc} \frac{1}{A(n-1)} & \frac{-1}{An(n-1)}\\ \frac{-1}{An(n-1)} & \frac{1}{n^{2}}\left(\frac{{\left(1+n{\hat{\sigma }}_{A}^{2}/{\hat{\sigma }}_{\epsilon }^{2}\right)}^{2}}{A-1}+\frac{1}{A(n-1)}\right) \end{array}\right).$$

It follows that

$$\;\mathrm{Var}\left({\hat{\sigma }}_{\epsilon }^{2}\right)=\frac{2{\text{MSE}}^{2}}{A(n-1)},\;\text{Cov}\left({\hat{\sigma }}_{\epsilon }^{2},{\hat{\sigma }}_{A}^{2}\right)=\frac{-2{\text{MSE}}^{2}}{An(n-1)},$$

$$\mathrm{Var}\left({\hat{\sigma }}_{A}^{2}\right)=\frac{2{\text{MSE}}^{2}}{n^{2}}\left(\frac{1}{A-1}+\frac{2(\text{MSA}-\text{MSE})/\text{MSE}}{A-1}+\frac{{\left(\text{MSA}-\text{MSE}\right)}^{2}/{\text{MSE}}^{2}}{A-1}+\frac{1}{A(n-1)}\right).$$

Therefore,

$$\begin{array}{ll} & \mathrm{Var}\left({\hat{\sigma }}_{\epsilon }^{2}\right)+2\text{Cov}\left({\hat{\sigma }}_{A}^{2},{\hat{\sigma }}_{\epsilon }^{2}\right)+\mathrm{Var}\left({\hat{\sigma }}_{A}^{2}\right)\\ & =\frac{2{\text{MSE}}^{2}}{A(n-1)}-\frac{4{\text{MSE}}^{2}}{An(n-1)}+\frac{2{\text{MSE}}^{2}}{n^{2}(A-1)}+\frac{4\text{MSE}(\text{MSA}-\text{MSE})}{n^{2}(A-1)}+\frac{2{\left(\text{MSA}-\text{MSE}\right)}^{2}}{n^{2}(A-1)}+\frac{2{\text{MSE}}^{2}}{n^{2}A(n-1)}\\ & =\frac{2}{n^{2}(A-1)}{\text{MSA}}^{2}+\frac{2{\left(1-1/n\right)}^{2}}{A(n-1)}{\text{MSE}}^{2}. \end{array}$$

The degrees of freedom (A1) and (A2) are then equal: *r*=*r* *′*.

## References

[1] Kenett RS , Zacks S , Amberti D . Modern Industrial Statistics: With Applications in R, MINITAB and JMP. 2nd ed Chichester, UK:Wiley & Sons; 2014.

[2] Dong X , Tsong Y , Shen M , Zhong J . Using tolerance intervals for assessment of pharmaceutical quality. J Biopharm Stat. 2015;25:317‐327. 25356617 10.1080/10543406.2014.972512

[3] Fuchs C , Kenett RS . Multivariate tolerance regions and f‐tests. J Qual Technol. 1987;19(3):122‐131. 10.1080/00224065.1987.11979053

[4] Francq B , Govaerts B . How to regress and predict in a Bland‐Altman plot? Review and contribution based on tolerance intervals and correlated‐errors‐in‐variables models. Statist Med. 2016;35:2328‐2358. 10.1002/sim.6872 26822948

[5] Choudhary P . A tolerance interval approach for assessment of agreement in method comparison studies with repeated measurements. J Stat Plan Inference. 2008;138:1102‐1115.

[6] Krishnamoorthy K , Mathew T . One‐sided tolerance intervals in balanced and unbalanced one‐way random models based on generalized confidence intervals. Technometrics. 2004;46(1):44‐52.

[7] Hoffman D , Kringle R . Two‐sided tolerance intervals for balanced and unbalanced random effects models. J Biopharm Stat. 2005;15(2):283‐293. 15796296 10.1081/BIP-200048826

[8] Mee R . *β*‐expectation and *γ*‐content tolerance limits for balanced one‐way ANOVA random model. Technometrics. 1984;26:251‐254.

[9] Liao C , Lin T , Iyer H . One‐ and two‐sided tolerance intervals for general balanced mixed models and unbalanced one‐way random models. Technometrics. 2005;47(3):323‐335.

[10] Sharma G , Mathew T . One‐sided and two‐sided tolerance intervals in general mixed and random effects models using small‐sample asymptotics. J Am Stat Assoc. 2012;107(497):258‐267.

[11] Howe W . Two‐sided tolerance limits for normal populations, some improvements. J Am Stat Assoc. 1969;64(326):610‐620.

[12] Chew V . Confidence, prediction, and tolerance regions for the multivariate normal distribution. J Am Stat Assoc. 1966;61(315):605‐617.

[13] Meeker W , Hahn G , Escobar L . Statistical Intervals: Guide for Practitioners and Researchers. 2nd ed Hoboken, NJ:Wiley & Sons; 2017 Wiley Series in Probability and Statistics.

[14] Brown H , Prescott R . Applied Mixed Models in Medicine. 1st ed North Scituate, MA: Duxbury Press; 1999.

[15] Qu L , Guennel T , Marshall S . Linear score tests for variance components in linear mixed models and applications to genetic association studies. Biometrics. 2013;69:883‐892. 24328714 10.1111/biom.12095

[16] Wolfinger R , Tobias R , Sall J . Computing Gaussian likelihoods and their derivatives for general linear mixed models. SIAM J Sci Comput. 1994;15(6):1294‐1310.

[17] Harville D . Maximum likelihood approaches to variance component estimation and to related problems. J Am Stat Assoc. 1997;72:320‐338.

[18] McLean R , Sanders W . Approximating degrees of freedom for standard errors in mixed linear models. In: Proceedings of the Statistical Computing Section; 1988; Alexandria, VA.

[19] Kackar R , Harville D . Approximations for standard errors of estimators of fixed and random effect in mixed linear models. J Am Stat Assoc. 1984;79(388):853‐862.

[20] Prasad N , Rao S . The estimation of mean squared error of small‐area estimators. J Am Stat Assoc. 1990;85:163‐171.

[21] Huber P . The behavior of maximum likelihood estimates under nonstandard conditions. In: Proceedings of the Fifth Berkeley Symposium on Mathematical Statistics and Probability; 1967; Berkeley, CA.

[22] White H . A heteroskedasticity‐consistent covariance matrix estimator and a direct test for heteroskedasticity. Econometrica. 1980;48:817‐838.

[23] Liang K‐Y , Zeger S . Longitudinal data analysis using generalized linear models. Biometrika. 1986;73:13‐22.

[24] Diggle P , Heagerty P , Liang K‐Y , Zeger S . Analysis of Longitudinal Data. 2nd ed Oxford, UK:Oxford University Press; 2002.

[25] Efron B , Hinkley D . Assessing the accuracy of the maximum likelihood estimator: Observed versus expected Fisher information. Biometrika. 1978;65(3):457‐487.

[26] Wand M . Fisher information for generalised linear mixed models. J Multivar Anal. 2007;98:1412‐1416.

[27] Searle S , Casella G , McCulloch C . Variance Components. Hoboken, NJ:Wiley & Sons; 1992 Wiley Series in Probability and Statistics.

[28] Sahai H , Ojeda M . Analysis of Variance for Random Models, Volume 2: Unbalanced Data ‐ Theory, Methods, Applications, and Data Analysis. Boston, MA:Birkhäuser; 2004 Analysis of Variance for Random Models.

[29] Kenward M , Roger J . Small sample inference for fixed effects from restricted maximum likelihood. Biometrics. 1997;53:983‐997. 9333350

[30] Francq B , Cartiaux O . Delta method and bootstrap in linear mixed models to estimate a proportion when no event is observed: application to intralesional resection in bone tumor surgery. Statist Med. 2016;35:3563‐3582. 10.1002/sim.6939 26990871

[31] Satterthwaite F . An approximate distribution of estimates of variance components. Biom Bull. 1946;2:110‐114. 20287815

[32] Graybill F , Wang C‐M . Confidence intervals on nonnegative linear combinations of variances. J Am Stat Assoc. 1980;75(372):869‐873.

[33] Ting N , Burdick R , Graybill F , Jeyaratnam S , Lu T‐FC . Confidence intervals on linear combinations of variance components that are unrestricted in sign. J Stat Comput Simul. 1990;35(3:4):135‐143.

[34] Shmueli G . To explain or to predict? Stat Sci. 2010;25(3):289‐310.

[35] SAS Institute Inc. JMP_*®*_ 14 Fitting Linear Models. Cary, NC: SAS Institute Inc; 2018.

[36] Qu L . Varcomp: Variance component models. R package version 0.1‐360. 2015 http://CRAN.R-project.org/package=varComp

[37] Cartiaux O , Banse X , Paul L , Francq B , Aubin C , Docquier P . Computer‐assisted planning and navigation improves cutting accuracy during simulated bone tumor surgery of the pelvis. Comput Aided Sur. 2013;18(1‐2):19‐26. 10.3109/10929088.2012.744096 23176154

[38] Cartiaux O , Paul L , Francq B , Banse X , Docquier P‐L . Improved accuracy with 3d planning and patient‐specific instruments during simulated pelvic bone tumor surgery. Ann Biomed Eng. 2014;42(1):205‐213. 23963884 10.1007/s10439-013-0890-7

[39] Cartiaux O , Jenny J‐Y , Joskowicz L . Accuracy of computer‐aided techniques in orthopaedic surgery: How can it be defined, measured experimentally, and analyzed from a clinical perspective? J Bone Joint Surg Am. 2017;99(8):e39. 28419041 10.2106/JBJS.15.01347

[40] Hoffman D . One‐sided tolerance limits for balanced and unbalanced random effects models. Technometrics. 2010;52(3):303‐312.

