# Supplementary material for: Confidence, prediction, and tolerance in linear mixed models
Source: Stat Med. 2019 Oct 28;38(30):5603–22. doi: 10.1002/sim.8386 (PMC6916346; doi:10.1002/sim.8386)
Supplement: Supplementary file 1 — SIM_8386‐Supp‐0001‐PI_MixedModels_Supp.pdf [file SIM-38-5603-s001.pdf]

Confidence, Prediction and Tolerance in Linear Mixed Models

Bernard G. Francq[1], Dan Lin, Walter Hoyer

Supplementary Information

This document provides additional figures, R and SAS code, and details to the manuscript.

Additional figures to Section 4 - (Average) Widths of the confidence, prediction and tolerance interval

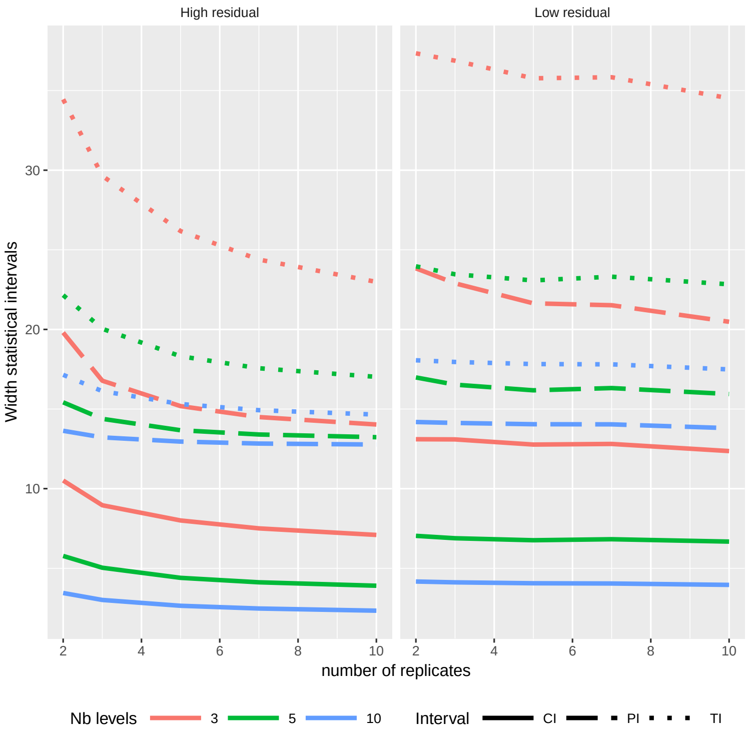

Figure S.1: Widths of the Confidence, Prediction and Tolerance Intervals in one random variable model, according to different sample sizes.

[1]bernard.x.francq@gsk.com, bernard.g.francq@uclouvain.be

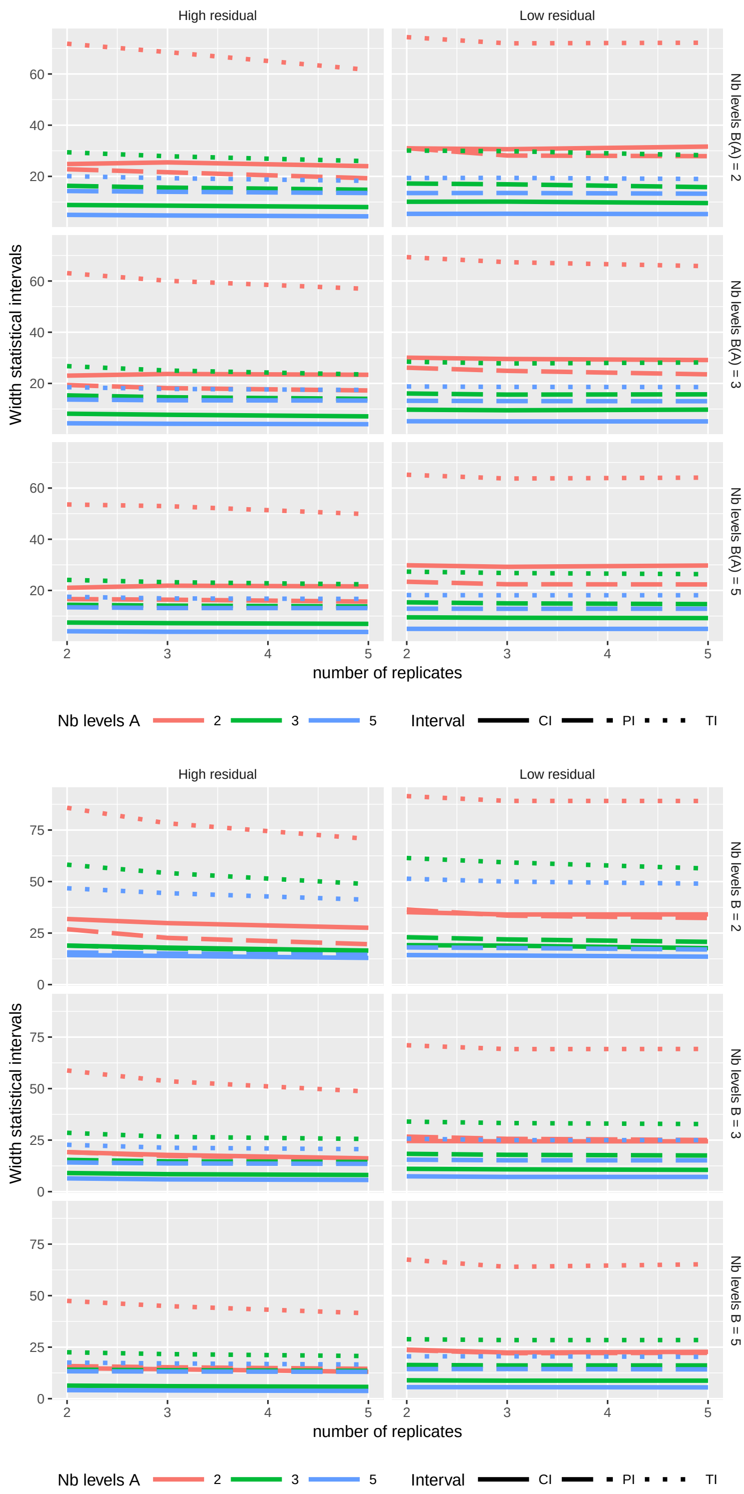

Figure S.2: Widths of the Confidence, Prediction and Tolerance Intervals in two nested (top) or two crossed (bottom) random variables model, according to different sample sizes.

Additional material to Section 4.4 - A paradox with confidence interval larger than prediction interval

Two nested random variables

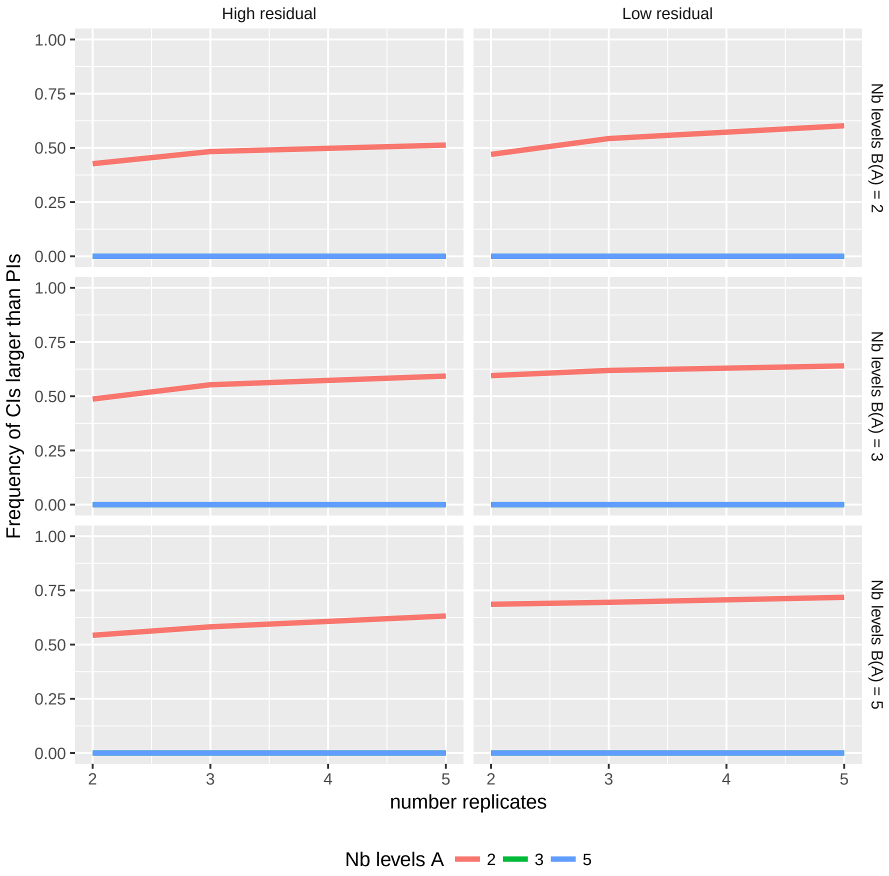

Figure S.3: Frequency of Confidence Intervals larger than Prediction Intervals in two nested random variables model, according to different sample sizes.

Two crossed random variables

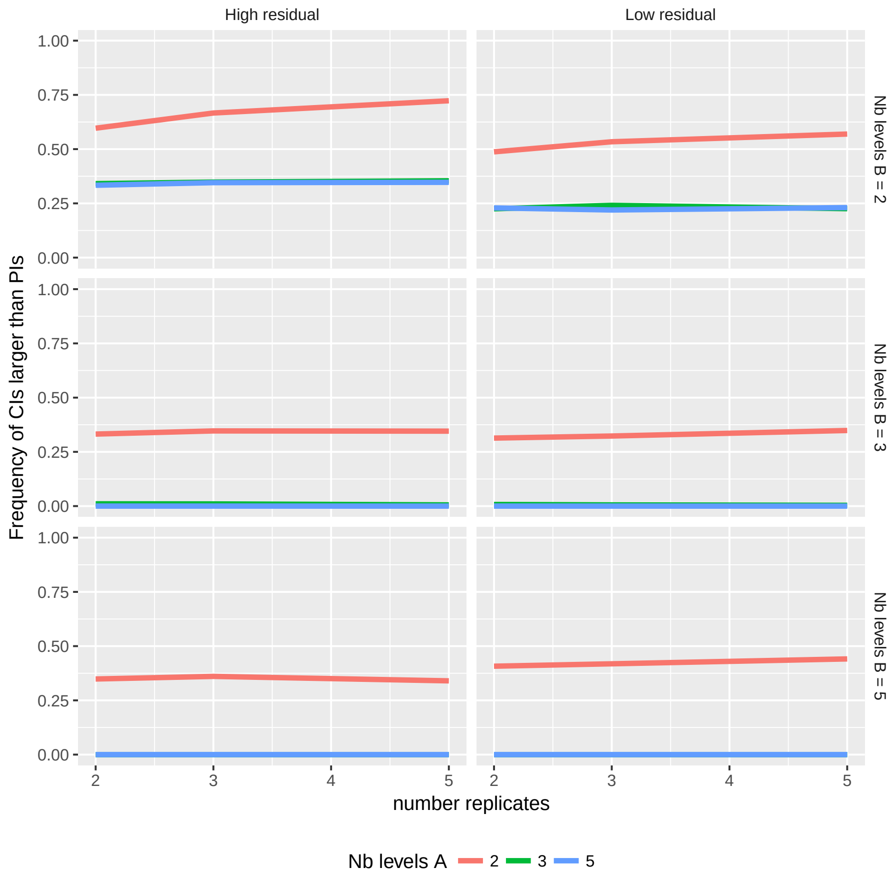

Figure S.4: Frequency of Confidence Intervals larger than Prediction Intervals in two crossed random variables model, according to different sample sizes.

Orthopedic Surgery Study, Intralesional Resection Risk - Section 5.1

Orthopedic Surgery Study, Intralesional Resection Risk - Data

Table 1: Error on Safe Margin (ESM) data with 23 Surgeons (S) (10 senior (T = 1) and 13 junior (T = 2)), 2 Procedures (1 = free hand, 2 = navigated), 4 Slices (Sl)

S	T	P	Sl	ESM	S	T	P	Sl	ESM
1	1	1	1	-0.199433025	12	2	2	1	3.645450552
1	1	1	2	-3.257110126	12	2	2	2	2.653294657
1	1	1	3	-6.097795531	12	2	2	3	1.176665963
1	1	1	4	-1.801171635	12	2	2	4	-0.556436938
1	1	2	1	2.47920259	13	2	1	1	6.995088621
1	1	2	2	0.556467843	13	2	1	2	4.50804089
1	1	2	3	-4.200284644	13	2	1	3	1.706578724
1	1	2	4	-0.503255249	13	2	1	4	-6.948706763
2	1	1	1	0.579324004	13	2	2	1	1.655868771
2	1	1	2	1.795684047	13	2	2	2	4.001138681
2	1	1	3	-7.84372224	13	2	2	3	1.859375282
2	1	1	4	-10.06203038	13	2	2	4	1.177417998
2	1	2	1	-2.12002035	14	2	1	1	-0.303659036
2	1	2	2	1.961510909	14	2	1	2	0.032991042
2	1	2	3	2.053270279	14	2	1	3	-8.837578381
2	1	2	4	4.28509354	14	2	1	4	5.272748506
3	1	1	1	0.594384847	14	2	2	1	0.332346267
3	1	1	2	13.08583217	14	2	2	2	0.028241458
3	1	1	3	-7.755593652	14	2	2	3	-1.28059761
3	1	1	4	-7.652534463	14	2	2	4	-1.091097724
3	1	2	1	2.186481394	15	2	1	1	-8.00357811
3	1	2	2	-0.253338241	15	2	1	2	-1.398753195
3	1	2	3	-2.68651742	15	2	1	3	-3.388708179
3	1	2	4	1.222242781	15	2	1	4	-10.4677522
4	1	1	1	7.008679635	15	2	2	1	-0.850897936
4	1	1	2	9.396533372	15	2	2	2	0.249953261
4	1	1	3	-2.119601508	15	2	2	3	-0.359438631
4	1	1	4	-3.948526636	15	2	2	4	0.276717839
4	1	2	1	1.969926726	16	2	1	1	-1.190984659
4	1	2	2	1.345195294	16	2	1	2	0.985835269
4	1	2	3	-0.845207026	16	2	1	3	-2.829419859
4	1	2	4	-2.315714263	16	2	1	4	-3.17059446
5	1	1	1	9.469642722	16	2	2	1	0.545197278
5	1	1	2	2.550111058	16	2	2	2	3.393442227
5	1	1	3	4.732536916	16	2	2	3	-0.475404056
5	1	1	4	5.394352277	16	2	2	4	1.048609558
5	1	2	1	0.666808812	17	2	1	1	2.939809264
5	1	2	2	4.740998052	17	2	1	2	10.85359371
5	1	2	3	-0.672978867	17	2	1	3	-3.722642625
5	1	2	4	-0.199835394	17	2	1	4	0.69149394

Continued on next page

Table 1 – *Continued from previous page*

S	T	P	Sl	ESM	S	T	P	Sl	ESM
6	1	1	1	-6.005567639	17	2	2	1	-0.49561995
6	1	1	2	8.732595809	17	2	2	2	1.318456583
6	1	1	3	-4.998981543	17	2	2	3	-1.363158874
6	1	1	4	5.347002874	17	2	2	4	1.501276621
6	1	2	1	5.265503882	18	2	1	1	-0.731155605
6	1	2	2	0.90179087	18	2	1	2	3.035331218
6	1	2	3	-0.982043294	18	2	1	3	10.79346831
6	1	2	4	-0.063627675	18	2	1	4	2.340906825
7	1	1	1	2.178684206	18	2	2	1	1.080578996
7	1	1	2	6.207547426	18	2	2	2	-4.322238012
7	1	1	3	-0.372947673	18	2	2	3	-4.037473506
7	1	1	4	-2.515639846	18	2	2	4	2.821756483
7	1	2	1	1.947725446	19	2	1	1	-7.637179919
7	1	2	2	2.742861691	19	2	1	2	-4.684496483
7	1	2	3	-1.10561243	19	2	1	3	-9.61826304
7	1	2	4	1.142331658	19	2	1	4	-10.88681822
8	1	1	1	3.473064383	19	2	2	1	0.528877263
8	1	1	2	6.834012012	19	2	2	2	5.000027971
8	1	1	3	0.319412923	19	2	2	3	5.036821017
8	1	1	4	-8.148487852	19	2	2	4	1.220096839
8	1	2	1	-0.573665795	20	2	1	1	-11.05336479
8	1	2	2	0.050016286	20	2	1	2	-0.571742129
8	1	2	3	-4.41972017	20	2	1	3	-11.6323534
8	1	2	4	-4.60992034	20	2	1	4	-5.553300133
9	1	1	1	3.227905411	20	2	2	1	-1.069254728
9	1	1	2	6.950558186	20	2	2	2	-1.822197379
9	1	1	3	-1.751515803	20	2	2	3	-0.796320339
9	1	1	4	-2.632273349	20	2	2	4	1.067784422
9	1	2	1	6.500339875	21	2	1	1	-1.695086081
9	1	2	2	1.446851398	21	2	1	2	2.676954209
9	1	2	3	-2.818420161	21	2	1	3	0.059231146
9	1	2	4	0.649838638	21	2	1	4	-0.030720039
10	1	1	1	-0.785676311	21	2	2	1	0.534649542
10	1	1	2	0.907771737	21	2	2	2	0.891207214
10	1	1	3	-1.314186537	21	2	2	3	0.510001948
10	1	1	4	-0.822383964	21	2	2	4	-0.578939074
10	1	2	1	1.960098655	22	2	1	1	1.882796607
10	1	2	2	-0.735292122	22	2	1	2	2.206442557
10	1	2	3	-0.192647106	22	2	1	3	0.165363349
10	1	2	4	-0.732942363	22	2	1	4	8.449809985
11	2	1	1	0.783003671	22	2	2	1	0.283382291
11	2	1	2	3.551837181	22	2	2	2	0.710908822
11	2	1	3	-4.417912977	22	2	2	3	-1.229336454
11	2	1	4	4.221018801	22	2	2	4	0.227583336
11	2	2	1	0.380875478	23	2	1	1	-1.565415884
11	2	2	2	3.128745017	23	2	1	2	1.85562997
11	2	2	3	-1.018740063	23	2	1	3	3.347694472
11	2	2	4	-1.762189915	23	2	1	4	4.237200596
12	2	1	1	2.661981324	23	2	2	1	2.803884792

Continued on next page

Table 1 – *Continued from previous page*

S	T	P	Sl	ESM	S	T	P	Sl	ESM
12	2	1	2	4.909653675	23	2	2	2	1.465135911
12	2	1	3	-1.798135049	23	2	2	3	-2.591379572
12	2	1	4	-5.352090715	23	2	2	4	0.171340156

Orthopedic Surgery Study, Intralesional Resection Risk - SAS Code

The data set is in the stacked format and named 'ESM'. The following SAS code is for the 'free hand' procedure (procedure = 1). The same code can be run for the navigated technology (procedure = 2).

```
/* Orthopedic Surgery Study, Intralesional Resection Risk */
/* Bernard G FRANCQ */

/* Estimate the Mixed Model */
ODS OUTPUT AsyCov = AsyCov SolutionF = SolutionF CovParms = CovParms ;
PROC MIXED DATA = ESM ASYCOV METHOD=REML;
CLASS decoupe operateur PROCEDURE;
MODEL ESM = decoupe / S CL DDFM = KENWARDROGER NOINT;
RANDOM operateur;
WHERE procedure = 1;
RUN;

/* Check Degrees of freedom and EMS for the tolerance intervals */
PROC GLM DATA = ESM;
CLASS decoupe operateur PROCEDURE;
MODEL ESM = decoupe operateur / NOINT;
RANDOM operateur;
WHERE procedure = 1;
RUN;

/* Calculate the Total Variance */
PROC TRANSPOSE DATA = CovParms OUT = CovParmsbis;
VAR Estimate;
RUN;
DATA CovParmsbis;
SET CovParmsbis;
varsurgeon = COL1;
varres = COL2;
vartot = varsurgeon + varres;
DROP _NAME_ COL1 COL2;
RUN;

/* Variances of variances */
DATA Asycov1 (RENAME = (CovP1 = varvarsurgeon CovP2 = covsurgeonres));
SET Asycov;
WHERE Row = 1;
DROP Row CovParm;
RUN;
DATA Asycov2 (RENAME = (CovP2 = varvarres));
SET Asycov;
WHERE Row = 2;
DROP Row CovParm CovP1;
RUN;
DATA Asycovbis;
SET Asycov1;
SET Asycov2;
```

```
varvartot = varvarsurgeon + varvarres + 2*covsurgeonres;
RUN;
PROC DELETE LIB = WORK DATA = Asycov1 Asycov2;
RUN;

    DATA var;
SET CovParmsbis;
SET Asycovbis (KEEP = varvartot);
RUN;

    /* merge (Confidence Intervals given by CI1 and CI2) */

    DATA Results;
SET Solutionf (DROP = tValue Probt Alpha RENAME = (Lower = CI1 Upper = CI2));
IF _N_=1 THEN SET var;
RUN;

    /* Calculate the Prediction and Tolerance Intervals */

    DATA Results (DROP = varvartot MSsurgeon tPI ksurgeon kres Hsurgeon Hres all);
SET Results;

    varPI = StdErr ** 2 + vartot;
MSsurgeon = varres + 4*varsurgeon;

    /* Calculate the degrees of freedom from the total variance */
dfvartot = 2 * (vartot ** 2 / varvartot);

    /* Formula (21) - 95% Prediction Interval */
tPI = quantile('T',.975,dfvartot);
PI1 = Estimate - tPI * sqrt(varPI);
PI2 = Estimate + tPI * sqrt(varPI);

    /* Formulae (26,27,28) - 95%-80% Tolerance Interval */

    ksurgeon = 1/4;
kres = 1-1/4;
Hsurgeon = (23-1)/quantile('CHISQUARE',0.2,23-1) - 1;
Hres = 66/quantile('CHISQUARE',0.2,66) - 1;
all = Hsurgeon**2 * ksurgeon**2 * MSsurgeon**2 + Hres**2 * kres**2 * varres**2;
TI1 = Estimate - quantile('NORMAL', 0.975) * sqrt(varPI) * sqrt(1+1/vartot*sqrt(all));
TI2 = Estimate + quantile('NORMAL', 0.975) * sqrt(varPI) * sqrt(1+1/vartot*sqrt(all));
RUN;
```

Orthopedic Surgery Study, Intralesional Resection Risk - R Code

```
# Orthopedic Surgery Study, Intralesional Resection Rik
# Estimate Mixed Model and calculate the different statistical intervals
# Bernard G FRANCQ

rm(list = ls())

ESM = data.frame(read.table(...))
ESM = ESM[ESM$PROCEDURE == 1,]
ESM$Surgeon = as.factor(ESM$Surgeon)
ESM$SLICE = as.factor(ESM$SLICE)

# Mixed Model

model = varComp(data = ESM, fixed = as.formula("ESM ~SLICE -1"), random = as.formula("~Surgeon"))
summary(model)

# Intercept and its standard error, and 95% Confidence Interval:

res = as.data.frame(attr(unclass(fixef(model)), "anova"))
res = data.frame(Estimate = coef(model), res[, c("Std. Error", "Lower", "Upper", "Df")])

# Variance components and their covariance matrix:

var.surgeon = model$varComp
var.res = model$sigma2
cov.matrix = vcov(model, what = "random")

# Total variance and its variance:

var.tot = var.surgeon + var.res
var.var.tot = sum(cov.matrix)

# Degrees of freedom for prediction

r = 2*var.tot^2/var.var.tot

# 95% Prediction Interval and 95%-80% Tolerance Interval:

res$dfPI = r
res$PI1 = res$Estimate - qt(0.975, r) * sqrt(res$Std..Error^2 + var.tot)
res$PI2 = res$Estimate + qt(0.975, r) * sqrt(res$Std..Error^2 + var.tot)

H.surgeon = 22 / qchisq(0.2, 22) - 1
H.res = 66 / qchisq(0.2, 66) - 1
comp.surgeon = H.surgeon^2 * (1/4)^2 * (4*var.surgeon + var.res)^2
comp.res = H.res^2 * (1-1/4)^2 * var.res^2
term = 1 + 1/var.tot * sqrt(comp.surgeon + comp.res)

res$TI1 = res$Estimate - qnorm(0.975) * sqrt(res$Std..Error^2 + var.tot) * sqrt(term)
res$TI2 = res$Estimate + qnorm(0.975) * sqrt(res$Std..Error^2 + var.tot) * sqrt(term)
```

Assay Validation Study - SAS Code

```
/* SAS Code - Assay Validation, Data from Hoffman and Kringle (2005) */
/* Bernard G FRANCQ */

data assay;
input run rep conc ;
cards;
1 1 0.969
1 2 0.976
1 3 0.938
2 1 0.952
2 2 0.993
2 3 0.956
3 1 0.989
3 2 0.883
3 3 0.981
4 1 1
4 2 0.969
4 3 0.954
5 1 0.959
5 2 0.989
5 3 1.02
6 1 1.02
6 2 1.09
6 3 1.02
;
run;

ODS OUTPUT AsyCov = AsyCov SolutionF = SolutionF CovParms = CovParms ;
PROC MIXED DATA = assay ASYCOV METHOD=REML;
CLASS run;
MODEL conc = / S CL DDFM = KENWARDROGER;
RANDOM run ;
RUN;

/* Check Degrees of freedom and EMS for the tolerance intervals */
PROC GLM DATA = assay;
CLASS run;
MODEL conc = run;
RANDOM run ;
RUN;

/* Calculate the Total Variance */
PROC TRANSPOSE DATA = CovParms OUT = CovParmsbis;
VAR Estimate;
RUN;

DATA CovParmsbis;
SET CovParmsbis;
varrun = COL1;
varres = COL2;
```

```
vartot = varrun + varres;
DROP _NAME_ COL1 COL2;
RUN;

    /* Variances of variances */
DATA Asycov1 (RENAME = (CovP1 = varvarrun CovP2 = covrunres));
SET Asycov;
WHERE Row = 1;
DROP Row CovParm;
RUN;
DATA Asycov2 (RENAME = (CovP2 = varvarres));
SET Asycov;
WHERE Row = 2;
DROP Row CovParm CovP1;
RUN;
DATA Asycovbis;
SET Asycov1;
SET Asycov2;
varvartot = varvarrun + varvarres + 2*covrunres;
RUN;
PROC DELETE LIB = WORK DATA = Asycov1 Asycov2;
RUN;

    DATA var;
SET CovParmsbis;
SET Asycovbis (KEEP = varvartot);
RUN;

    /* merge (Confidence Intervals given by CI1 and CI2) */

    DATA Results;
SET Solutionf (DROP = tValue Probt Alpha RENAME = (Lower = CI1 Upper = CI2));
SET var;
RUN;

    /* Calculate the Prediction and Tolerance Intervals */
DATA Results (DROP = varvartot MSrun tPI krun kres Hrun Hres all);
SET Results;

    varPI = StdErr ** 2 + vartot;
MSrun = varres + 3*varrun;

    /* Calculate the degrees of freedom from the total variance */
dfvartot = 2 * (vartot ** 2 / varvartot);

    /* Formula (21) paper - 95% Prediction Interval */
tPI = quantile('T',.975,dfvartot);
PI1 = Estimate - tPI * sqrt(varPI);
PI2 = Estimate + tPI * sqrt(varPI);

    /* Formulae (26,27,28) paper - 95%-90% Tolerance Interval */
```

```
krun = 1/3;
kres = 1-1/3;
Hrun = (6-1)/quantile('CHISQUARE',0.1,6-1) - 1;
Hres = 12/quantile('CHISQUARE',0.1,12) - 1;
all = Hrun**2 * krun**2 * MSrun**2 + Hres**2 * kres**2 * varres**2;
TI1 = Estimate - quantile('NORMAL', 0.975) * sqrt(varPI) * sqrt(1+1/vartot*sqrt(all));
TI2 = Estimate + quantile('NORMAL', 0.975) * sqrt(varPI) * sqrt(1+1/vartot*sqrt(all));
RUN;
```

Assay Validation Study - R Code

```
# Assay Validation Study, data from Hoffman and Kringle (2005)
# Estimate Mixed Model and calculate the different statistical intervals
# Bernard G FRANCQ

rm(list = ls())

library(varComp) # Archived R package

run = rep(1:6, each = 3)
rep = rep(1:3, length.out = 18)
y = c(0.969, 0.976, 0.938, 0.952, 0.993, 0.956, 0.989, 0.883, 0.981, 1, 0.969, 0.954, 0.959, 0.989, 1.02, 1.02, 1.09, 1.02)
dat = data.frame(run = run, rep = rep, y = y)
dat$run = as.factor(dat$run)

# Mixed Model

res = varComp(data = dat, fixed = as.formula("y ~1"), random = as.formula("~run"))
summary(res)

# Intercept and its standard error, and 95% Confidence Interval:

estimate = unname(coef(res, what = "fixed"))
stderr.estimate = attr(unclass(fixef(res)), "anova")[1,1]
attr(unclass(fixef(res)), "anova")

# Variance components and their covariance matrix:

var.run = res$varComp
var.res = res$sigma2

cov.matrix = vcov(res, what = "random")

# Total variance and its variance:

var.tot = unname(var.run + var.res)
var.var.tot = sum(cov.matrix)

# Degrees of freedom for prediction:

r = 2var.tot^2/var.var.tot

# 95% Prediction Interval and 95%-90% Tolerance Interval:

PI1 = estimate - qt(0.975, r) * sqrt(stderr.estimate^2 + var.tot)
PI2 = estimate + qt(0.975, r) * sqrt(stderr.estimate^2 + var.tot)

H.run = 5 / qchisq(0.1, 5) - 1
H.res = 12 / qchisq(0.1, 12) - 1
comp.run = H.run^2 * (1/3)^2 * (3*var.run + var.res)^2
```

comp.res = H.res^2 * (1-1/3)^2 * var.res^2
term = 1 + 1/var.tot * sqrt(comp.run + comp.res)

TI1 = estimate - qnorm(0.975) * sqrt(stderr.estimate^2 + var.tot) * sqrt(term)
TI2 = estimate + qnorm(0.975) * sqrt(stderr.estimate^2 + var.tot) * sqrt(term)
